# scDIAGRAM: detecting chromatin compartments from individual single-cell Hi-C matrix without imputation or reference features

**DOI:** 10.1093/bib/bbag096

**Published:** 2026-03-08

**Authors:** Yongli Peng, Yujing Deng, Menghan Liu, Zhiyuan Liu, Ya-Hui Li, Xiang-Yu Zhao, Dong Xing, Jinzhu Jia, Hao Ge

**Affiliations:** Beijing International Center for Mathematical Research (BICMR), Peking University, No.5 Yiheyuan Road, Haidian District, Beijing 100871, China; Biomedical Pioneering Innovation Center (BIOPIC), Peking University, No.5 Yiheyuan Road, Haidian District, Beijing 100871, China; Biomedical Pioneering Innovation Center (BIOPIC), Peking University, No.5 Yiheyuan Road, Haidian District, Beijing 100871, China; Biomedical Pioneering Innovation Center (BIOPIC), Peking University, No.5 Yiheyuan Road, Haidian District, Beijing 100871, China; Peking University Institute of Hematology, National Clinical Research Center for Hematologic Disease, Peking University People's Hospital, No.11 Xizhimen South Street, Xicheng District, Beijing 100044, China; Peking University Institute of Hematology, National Clinical Research Center for Hematologic Disease, Peking University People's Hospital, No.11 Xizhimen South Street, Xicheng District, Beijing 100044, China; Biomedical Pioneering Innovation Center (BIOPIC), Peking University, No.5 Yiheyuan Road, Haidian District, Beijing 100871, China; Beijing Advanced Innovation Center for Genomics, Peking University, No.5 Yiheyuan Road, Haidian District, Beijing 100871, China; School of Public Health and Center for Statistical Science, Peking University, No.5 Yiheyuan Road, Haidian District, Beijing 100871, China; Beijing International Center for Mathematical Research (BICMR), Peking University, No.5 Yiheyuan Road, Haidian District, Beijing 100871, China; Biomedical Pioneering Innovation Center (BIOPIC), Peking University, No.5 Yiheyuan Road, Haidian District, Beijing 100871, China

**Keywords:** single-cell Hi-C, A/B compartment, statistical modeling, chromatin heterogeneity

## Abstract

Single-cell Hi-C (scHi-C) provides unprecedented insight into 3D genome organization, but its sparse and noisy data pose challenges in accurately detecting A/B compartments, which are crucial for understanding chromatin structure and gene regulation. We presented scDIAGRAM, a data-driven method for annotating A/B compartments in single cells using direct statistical modeling and graph community detection. Unlike existing approaches, scDIAGRAM infers chromatin compartments directly from individual scHi-C matrix without imputation or external reference features, and subsequently assigns A/B labels using conventional genomic annotations. Accuracy and robustness of scDIAGRAM were illustrated through simulated scHi-C datasets and a human cell line. We applied scDIAGRAM to real scHi-C datasets from the mouse brain cortex, mouse embryonic development, and human acute myeloid leukemia, demonstrating its ability to capture compartmental shifts associated with transcriptional variation. This robust framework offers new insights into the functional roles of chromatin compartments at single-cell resolution across various biological contexts.

## Introduction

Advancements in 3D whole-genome mapping techniques, such as Hi-C, have significantly improved our understanding of genome organization within the nucleus [1–5]. Bulk Hi-C studies have revealed that the genome is organized into hierarchical structures, including chromosome territory [6], A/B compartments [1], topologically associating domains (TADs) [7, 8], and chromatin loops [9]. More recently, the integration of single-cell mapping technologies with conventional Hi-C has led to the emergence of single-cell Hi-C (scHi-C), allowing for the analysis of 3D genome organization at the resolution of individual cells [10–14].

However, scHi-C data are typically sparse and noisy, posing substantial challenges in understanding the variability of these structures at the single-cell level [15–19]. To address these challenges, computational tools have been developed, either by improving data quality through imputation [20–25] or by explicitly detecting multi-scale genome structures in individual cells without imputation [26–29].

In this work, we focused on annotating A/B compartments using only individual single-cell contact matrices. In bulk Hi-C analysis, compartmentalization is typically inferred from the normalized observed/expected (O/E) matrix or correlation matrices [1]. Principal component analysis (PCA) applied to the correlation matrix classifies the genome into open (A) and closed (B) compartments, corresponding to euchromatin and heterochromatin, respectively. This classification is further supported by fluorescence *in situ* hybridization experiments [30, 31]. Recently, tools such as cooltools [32] have streamlined this process for bulk Hi-C data.

Several computational methods were developed to identify single-cell compartments (scCompartments) in scHi-C data, often relying on external genomic features or imputation to address data sparsity. The A compartment was typically associated with higher gene density, greater CpG density, and stronger correlations with active histone modifications [1, 12, 33, 34]. Accordingly, one approach leveraged the CpG density from a reference genome combined with scHi-C matrices to infer A/B compartments in individual cells [12]. This method, referred as scA/B values, treated the Hi-C matrix as a graph and averaged CpG values across neighboring loci. This reference-guided strategy might produce annotations that reflect CpG patterns rather than the true 3D chromatin structures of single cells. Other methods focused on imputing missing contacts in sparse scHi-C matrices before applying compartment annotation techniques originally developed for bulk Hi-C like PCA [13, 20–22]. Some approaches, like Higashi [21, 22], and recently proposed scGHOST built upon Higashi [35], incorporated information from similar or neighboring cells to enhance imputation that could possibly blur biological differences and lead to an averaging effect. Even within-cell imputation approaches, such as that employed by scHiCluster [20], carried the risk of distorting compartment structures due to potential errors in imputed values. These challenges highlight the need for a method that directly analyzes scHi-C data without external dependencies, preserving both the integrity of chromatin structures and the biological variability across cells.

Here, we introduced scDIAGRAM (single-cell compartments annotation by DIrect stAtistical modeling and GRAph coMmunity detection), a novel computational tool designed to annotate chromatin A/B compartments in scHi-C data. The method addressed the challenges posed by sparse and noisy scHi-C datasets by applying direct statistical modeling and graph community detection [36, 37] to infer chromatin compartments directly from scHi-C interaction patterns, without relying on imputation or external reference features; A/B labels are subsequently assigned using conventional genomic annotations. scDIAGRAM was tested on both simulated and real datasets, including mouse brain cortex, mouse developing embryos, and human acute myeloid leukemia (AML) [38, 39], showing its ability to preserve compartmental heterogeneity and capture dynamic changes in genome organization. By linking compartmental shifts to transcriptional and epigenetic variations, scDIAGRAM enhanced our understanding of the functional roles of chromatin compartments and offered a robust framework for exploring 3D genome organization at single-cell resolution across diverse biological systems.

## Materials and methods

**Figure 1:**
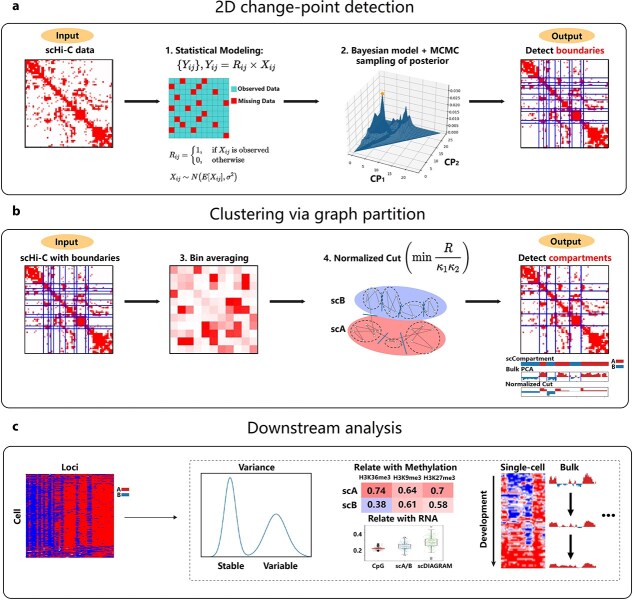
Schematic workflow of scDIAGRAM. (a) scDIAGRAM takes an scHi-C matrix as input, treating the problem as a 2D CP detection problem. A direct statistical model is established, and scDIAGRAM employs a Bayesian model with MCMC to detect CP positions. (b) Bin averaging is performed to each block formed by the detected CPs, and then a graph partitioning algorithm is applied to classify the genomic loci groups separated by CPs into two compartments. (c) The output is a heatmap representing the annotated compartments for each genomic loci. Each row corresponds to a cell, and each column represents a loci. The heatmap can be binary (a or b compartment) or real-valued (Ncut values), with A compartments and higher Ncut values indicating more active loci. Using scDIAGRAM, one can study compartmental heterogeneity within single cells, stable and variable genomic loci, the relationship between scCompartments with methylation and RNA expression, or analyze dynamic compartmental changes during development, among many other applications.

scDIAGRAM took an intrachromosomal Hi-C contact matrix as input, in which the genome was divided into discrete regions (i.e. “loci” or “bin”) at a specified resolution. The input data could be either bulk Hi-C or scHi-C matrices, with varying resolutions. The workflow began with 2D change-point (CP) detection, followed by the step of graph partitioning, to address the significant noise inherent in scHi-C data and annotate compartments for each loci. The underlying computational workflow was summarized in Fig. 1. Further details of the algorithm were provided below.

### 2D change-point detection

 We first constructed a direct statistical model for the contact matrix and framed the annotation as a 2D CP detection problem. We then applied Bayesian modeling and Markov Chain Monte Carlo (MCMC) methods to obtain the maximum likelihood estimation (MLE) of the problem.

For each measured contact matrix $\{Y_{ij}\}$, in which $Y_{ij}$ is the measured contact number between the $i$th and $j$th loci, we assumed that $Y_{ij} = R_{ij} \times X_{ij}$, where $R_{ij}$ represented the dropout effect, and $X_{ij}$ denoted the true contact number inside the cell. These variables were assumed to be independent of each other.

The distributions of $X_{ij}$ is assumed to be Gaussian, i.e. $X_{ij} \sim N(E[X_{ij}], \sigma ^{2})$, and $R_{ij}$ follows a Bernoulli distribution. If $X_{ij}$ is observed, $R_{ij}=1$; and it is 0 otherwise.

We focused on detecting the compartment boundaries as CPs in the 2D contact matrix. Specifically, $K$ CPs divide the matrix into $(K+1) \times (K+1)$ blocks. We assumed that the parameters $P(R_{ij}=1)$ and $E[X_{ij}]$ were constant for all pairs $(i,j)$ within the same block. These parameters were represented by two $(K+1) \times (K+1)$ matrices, denoted as $\{r_{kl}\}_{k,l=1}^{K+1}$ and $\{\mu _{kl}\}_{k,l=1}^{K+1}$. We could solve the 2D CP detection problem using MLE.

Since each gap between adjacent loci could be considered as a CP, explicitly computing the MLE was computationally infeasible. Therefore, we turned to a Bayesian model and employed MCMC sampling to obtain samples of the parameters and then obtained the MLE.

First, we used one-hot encoding for the CP positions. We assumed that the dimension of contact matrix was $n$, which resulted in $(n-1)$ potential CP positions in total. The state space for CP positions was $\{0,1\}^{n-1}$, where $1$ indicated the presence of a CP at a given position and $0$ otherwise.

Next, we imposed priors on these states. We set $K$ CPs in total ($K$ is a hyperparameter and shall be determined in advance), then the prior was $\frac{1}{\binom{n-1}{K}}$ (uniform across states) and the posterior was proportional to the likelihood. In this way, the genomic loci ${1,2,\cdots ,n}$ were divided into $K+1$ groups, denoted as ${g_{1}, g_{2}, \cdots , g_{K+1}}$.

By Bayes’ formula, we had the posterior distribution 


\begin{align*} \textrm{Posterior}\propto& \prod_{k, l=1}^{K+1}r_{kl}^{S_{kl}}(1-r_{kl})^{N_{kl}-S_{kl}}\\ &\times \exp \left(-\frac{1}{2\sigma^{2}} \sum_{i, j=\mathbf{1}}^{n} (Y_{ij}-\mu_{kl})^{2}1_{i\in g_{k}}1_{j\in g_{l}}1_{Y_{ij}\neq 0}\right), \end{align*}

where $N_{kl}$ was the sample size of the $(k,l)$-block and $S_{kl}=\sum _{i\in g_{k}, j\in g_{l}}1_{X_{ij}\neq 0}$ was the nonzero sample size in the $(k,l)$-block. $\sigma ^{2}$, $\mu _{kl}$, and $r_{kl}$ needed to be estimated from the data once the positions of all CPs were given. For simplicity, we used the sample variance and the sample mean of nonzero entries.

Thus 


(1) \begin{align*} \textrm{Posterior}\propto& \prod_{k, l=1}^{K+1}\hat{r}_{kl}^{S_{kl}}(1-\hat{r}_{kl})^{N_{kl}-S_{kl}}\exp \left(-\frac{1}{2\hat{\sigma}^{2}} \sum_{k, l=1}^{K+1} S_{kl}V_{kl}\right)\end{align*}

where $\hat{r}_{kl} = \frac{S_{kl}}{N_{kl}}$, and $V_{kl}$ was the sample variance of nonzero entries inside the $(k, l)$-block.

We used Metropolis–Hasting (MH) methods to sample from the posterior distribution (or just the likelihood). See Supplementary Methods for details of the algorithm.

### Clustering of loci group via graph partition

Before graph partitioning, we accounted for the genomic distance-dependent decay of Hi-C contact frequencies. In bulk Hi-C data, this effect is typically addressed using observed-over-expected (O/E) normalization, and we followed standard practice by applying both 2D CP detection and graph partitioning on O/E-normalized matrices. In scHi-C data, however, direct O/E normalization is challenging due to extreme sparsity that can substantially amplify noise. To mitigate this issue, we adopted a modified strategy inspired by BandNorm [40], in which the expected contact frequency was estimated from a pseudo-bulk profile. Specifically, 2D CP detection was performed on the raw contact matrix, where distance effects have limited influence, followed by graph partitioning on the O/E-normalized data. In practice, O/E normalization was implemented using cooltools [32], and our modified pipeline is publicly available on GitHub.

With the positions of all $K$ CPs identified, all genomic loci were divided into $(K+1)$ groups. We employed a graph partitioning approach to cluster these groups into two compartments.

We treated each group of loci (bins) between two adjacent CPs as a node in a graph. The $K$ CPs divided the matrix into $(K+1)\times (K+1)$ blocks. We computed the average value of contact numbers within each block, as the weight for the edge connecting the two nodes, thereby reducing the original large ${n\times n}$ matrix into a smaller ${(K+1)\times (K+1)}$ one.

We applied a graph partitioning framework called normalized cut to the constructed weighted graph [36, 37], for its simplicity and effectiveness. It was formulated as an optimization problem $\min \frac{R}{\kappa _{1} \kappa _{2}}$, where $R$ represented the graph cut (the total number of contacts between A and B compartments) and $\kappa _{1},\kappa _{2}$ were the sums of degrees in the two clustered compartments.

This problem can be addressed using spectral methods. The second largest eigenvector $\nu _{2}$ of $\mathbf{D}^{-1/2}\mathbf{A}\mathbf{D}^{-1/2}$ is exactly a relaxed solution, in which $\mathbf{A}$ is the adjacency matrix and $\mathbf{D}$ is the diagonal matrix with elements $D_{ii} = k_{i}$. $k_{i}$ is the degree of node $i$. See Supplementary Methods for more details.

To obtain a discrete-valued vector for graph partitioning, $\nu _{2}$ was rounded, typically using $0$ as a threshold and nodes were assigned to the two compartments based on the sign of $\nu _{2}$ (according to Newman [36], the result was robust to the rounding strategy chosen for division). The compartmental value of each locus just inherited from the values of the corresponding node, i.e. the group of loci separated by CPs.

The remaining step is to add A/B annotation, i.e. determining which compartment is A or B. Based on bulk Hi-C analysis, we knew that the CpG density in the reference genome was strongly correlated with compartments, with the A compartment typically corresponding to regions of high CpG density. Following the convention in bulk Hi-C studies, we determined the sign by comparing the Pearson correlation between $\nu _{2}$ with the CpG density. A low absolute value of the correlation may indicate weak compartmentalization shown at this single cell.

### Hyperparameter settings

The number of CPs ($K$) was determined in a data-driven manner by assessing the stability of A/B compartment annotations produced by scDIAGRAM as $K$ increased. Specifically, compartment annotations obtained at successive values of $K$ were compared using correlation and intersection-based metrics. Candidate values of $K$ ranged from 10 to 200 in increments of 10.

$K$ was chosen for each dataset when the correlation between annotations at $K$ and $K+10$ exceeded 0.8 and their intersection exceeded 0.9, indicating that further increases in $K$ led to only marginal changes in compartment assignments. This criterion was applied consistently across all datasets, and the resulting values of $K$ are reported in Supplementary Fig. S1. Once determined at the dataset level, $K$ was fixed across all cells and cell types within that dataset to ensure comparability, while datasets representing distinct biological contexts (e.g. neurons versus developing embryos) were evaluated independently using the same procedure. We found this approach to be robust and adaptive across datasets.

For scHi-C datasets, we first determined the CP number for several cells and obtained the largest one among them. Then this CP number was fixed and applied to all other cells in this dataset. Typically, this value was set much higher than the CP number used for bulk Hi-C data. The choice of CP number also depended on the resolution; e.g. a reasonable choice was $\sim 100$ CPs at the resolution of 100 kilobase (kb) and $20$–$40$ CPs at 1 megabase (Mb). In this study, we mainly focused on scHi-C matrices at $100$ kb resolution. Comparable results could also be obtained at 1 Mb resolution, albeit with coarser structural details. We tested scDIAGRAM on a real single cell and found that the results remained stable as the number of CPs increased (Supplementary Fig. S2).

### Simulated scHi-C matrices

To benchmark the accuracy and robustness of scDIAGRAM, we generated simulated scHi-C datasets derived from imaging-based 3D structural models, bulk Hi-C matrices, and an Hi-C simulating method FreeHi-C [41]. These datasets were designed to reflect varying levels of sparsity and biological heterogeneity observed in real data. Details of the simulation protocols, parameter settings, and ground-truth annotations are provided in the Supplementary Methods.

Beyond heuristic downsampling strategies, several simulation tools have been developed to generate *in silico* Hi-C data for benchmarking. FreeHi-C [41] extends downsampling by learning interaction distributions from real Hi-C data, while scHi-CSim [42] is specifically designed to simulate scHi-C data with realistic sparsity and cell-to-cell heterogeneity. In this study, we focused on FreeHi-C for accuracy benchmarking, as it produces simulated data with well-defined compartment ground truth, which is conceptually closer to downsampling-based evaluations and facilitates direct comparison with methods such as PCA and scA/B.

### Real scHi-C and scRNA-seq datasets

Publicly available scHi-C datasets from mouse brains and mouse embryonic development were processed using standard pipelines. Contact matrices were normalized using cooler and cooltools, and pseudo-bulk matrices were constructed for each condition or cell type. We ran scDIAGRAM on each chromosome separately.

We collected AML patient samples and their pathological results from the Department of Hematology at Peking University People’s Hospital. All samples were obtained via bone marrow aspiration. The study was approved by the Ethics Committee of Peking University People’s Hospital (2024PHB391-001). All patients signed informed consent forms as required. The data processing method for AML in this study follows the same procedure as in [38].

scRNA-seq data were preprocessed with Seurat for clustering and marker genes were identified using Seurat [43] with default parameters.

See Supplementary Methods for more details on the real single-cell datasets.

### Comparing scDIAGRAM with other methods

We compared scDIAGRAM with scA/B values [12], compartments derived from imputed scHi-C matrices [20, 21] or 3D structure modeling [12], and two recent methods, MaxComp [25] and scGHOST [35].

The output of scA/B is only a real-valued compartmentalization. To obtain binary annotations, we applied rank/quantile normalization to transform these values into the range [0,1], and then binarized the compartments using a cutoff of 1/2. We used scHiCluster with default parameters [20] to impute the scHi-C matrices, then applied cooltools [32] on the imputed matrices to generate the scCompartments.

We trained Higashi without using neighboring cell information (0 nbr) [21] to impute the scHi-C matrices and applied its built-in method to call compartments at the single-cell level. Other parameters were set as default.

We also utilized the 3D modeling algorithm by the Hickit software with default parameters [12], on downsampled scHi-C datasets. Once the structure was generated, we took the inverse of the pairwise spatial distance matrices and applied cooltools to them. In this manner, we treated the 3D modeling as another method of imputing scHi-C matrices.

We applied MaxComp [25] to the DNA MERFISH imaging data from human IMR90 cells [44] that provides 3D coordinates and speckle distances for each genomic locus. Using the authors’ code, we constructed a graph from these spatial features and reformulated compartment annotation as a “Max-cut problem”, which was then solved using standard tools.

For scGHOST [35], we used Higashi-imputed scHi-C data along with scCompartments derived from Higashi. All default parameters were used. To make the results comparable to scDIAGRAM, scGHOST’s subcompartments (A1, A2, B1, B2, and B3) were merged into two groups (A/B).

#### Evaluation criteria

The methods were evaluated using several metrics, including correlation, intersection, and accuracy. Both Pearson and Spearman correlations were assessed. Intersection was quantified as the fraction of correctly assigned loci into A/B compartments for each chromosome in each cell. Accuracy at each loci was similarly defined as the fraction of correctly assigned samples among all samples, again focusing on loci classification.

### Stable and variable single-cell compartments

We computed the variance of binary compartments (1 for A and 0 for B) for each loci across all cells, with values ranging from 0 to 0.25. Genomic loci with stable and variable compartments were identified by applying a cutoff at the 50th percentile of this variance [24, 35]. To further explore the robustness of this classification, we also examined the top and bottom 25th, 10th, and 5th percentiles of the variance.

## Results

### scDIAGRAM is validated on simulated scHi-C matrices and a human cell line

We began by validating the performance of scDIAGRAM in comparison to scA/B and compartments obtained from scHiCluster, Higashi imputation, scGHOST, and MaxComp, using simulated scHi-C datasets and scHi-C data from a human cell line.

#### Simulation via downsampling pseudo-bulk Hi-C data

We first generated synthetic data through downsampling a pseudo-bulk Hi-C matrix of the Ex1 cell type on chr7 [38], with sample rates ranging from 1/400 to 1/3200 (Supplementary Methods). First of all, scDIAGRAM accurately predicted the correct compartments in the pseudo-bulk data itself, in comparison to that obtained using cooltools, with 0.94 intersection and 0.84 Pearson correlation. scA/B only had 0.825 intersection and 0.6 Pearson correlation (Supplementary Fig. S3).

**Figure 2:**
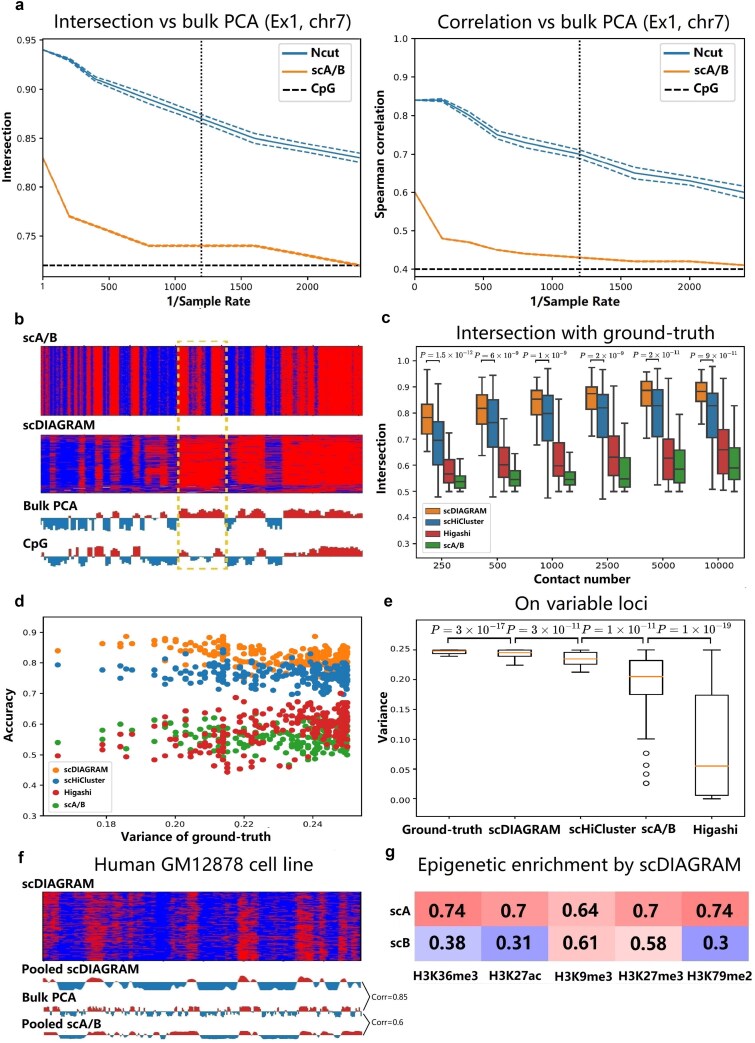
Evaluation of the performance of scDIAGRAM in simulated scHi-C dataset and a human cell line GM12878. (a) The intersection and Spearman correlation versus bulk PCA on a simulated scHi-C dataset via downsampling a pseudo-bulk Hi-C matrix. The vertical dash line is the sample rate of a typical real scHi-C dataset, and the horizontal dash line is the intersection or correlation between the CpG density and bulk PCA. (b) scDIAGRAM is more close to bulk PCA compared with scA/B, illustrated by the heatmap from the same downsampled data as (a). Each row represents a cell. As indicated in the boxed region, the CpG density is quite different from bulk PCA. (c and d) The intersection and accuracy on a simulated scHi-C dataset via downsampling a single-cell 3D imaging data. Accuracy is computed when the contact number is 1000, with genomic loci ordered by its compartmental variance calculated from ground-truth. The $P$-value $P=7\times 10^{-82}$ between the accuracy of scDIAGRAM and scHiCluster in (d). (e) scDIAGRAM is more heterogeneous compared with scHiCluster, Higashi, and scA/B. (f) Heatmap of scDIAGRAM and bulk PCA on human cell line GM12878. (g) Enrichment of epigenomic signals (average of these signals’ fold change) in pseudo-bulk compartments.

For downsampled data, scDIAGRAM showed closer alignment with the ground truth than scA/B, both for binary compartments (measured by intersection) and real-valued compartments (measured by Spearman correlation), across various sample rates (Fig. 2a, Supplementary Fig. S4A and B). Similar results were observed for chromosome $7$ of the mixed late mesenchyme cell type (Supplementary Fig. S4C), as well as for data simulated by FreeHi-C (Supplementary Fig. S5).

Moreover, at very low sample rates, where the data became highly sparse, scA/B often converged to the CpG signal, which is a universal feature independent of scHi-C data. In contrast, scDIAGRAM maintained better alignment with the pseudo-bulk data used to generate the simulated scHi-C matrices (Fig. 2a). This phenomenon was further highlighted by examining regions where the CpG signal and bulk PCA differed significantly (Fig. 2b).

#### Simulation via downsampling single-cell 3D genome imaging data

Next, we generated synthetic data by downsampling single-cell 3D genome imaging data [44], with contact numbers ranging from 250 to 10 000, which is comparable to real scHi-C datasets (Supplementary Methods, Supplementary Fig. S6). We used chromosome 2 and a dataset of 300 cells, similar to what has been done in [21].

We compared the performance of scDIAGRAM with scA/B, scHiCluster followed by PCA and Higashi with its built-in compartments (Fig. 2c, Supplementary Fig. S7A). scDIAGRAM outperformed scHiCluster in binary compartment annotation (measured by intersection) (Fig. 2c), while showing comparable performance in predicting real-valued compartments (measured by Pearson and Spearman correlation; Supplementary Fig. S7B). Additionally, we observed that the intersection and correlation from scHiCluster often exhibited larger variances, suggesting potential instability in the imputation. In contrast, scA/B performed poorly on downsampled data, both for binary and real-valued compartments (Fig. 2c).

Higashi performed slightly better than scA/B, but remained inferior to scDIAGRAM and scHiCluster. This discrepancy may stem from the pronounced heterogeneity inherent in the dataset. Higashi called scCompartments by projecting imputed matrices onto the pseudo-bulk compartment. As a result, it tends to produce relatively homogeneous compartments across cells (see below), suggesting that Higashi may be less suitable for datasets characterized by high heterogeneity.

With a fixed contact number, we evaluated the classification accuracy for each locus across different cells (Methods). In Fig. 2d, when the contact number was 1000, scDIAGRAM outperformed scHiCluster, Higashi, and scA/B in terms of accuracy, with scA/B showing the poorest performance. Each dot in the plot represents a genomic locus, ordered by the compartmental variance of the ground truth along the x-axis. This observation held universally for loci with different variances. The same results were observed for different contact numbers (Supplementary Fig. S7C).

Since 3D imaging data can generate a complete Hi-C matrix for each single cell, we were able to explore compartmental heterogeneity within this dataset. We computed the variance of binary scCompartment values across cells for each genomic loci and found that scDIAGRAM exhibited greater heterogeneity compared with scHiCluster, Higashi, and scA/B (Supplementary Fig. S7D), and was closer to the true heterogeneity directly obtained from the 3D imaging data. This difference was especially pronounced for the more variable loci (Fig. 2e).

We further compared scDIAGRAM with MaxComp, a recently proposed method that annotates A/B compartments from 3D chromosome structures derived from imaging data or reconstructed 3D models [25]. Unlike interaction-based approaches that operate on Hi-C contact matrices, MaxComp defines compartments from spatial distance graphs. As a result, the compartments inferred by MaxComp reflect a distance-based definition that is not strictly equivalent to interaction-based Hi-C-derived A/B compartments.

Using the same single-cell 3D imaging dataset in [44], we applied MaxComp, scDIAGRAM, and single-cell PCA (scPCA) to annotate A/B compartments for each cell (see Supplementary Methods for details). Overall, scDIAGRAM and MaxComp yielded broadly similar large-scale compartment structures across the genome. However, we observed systematic differences at the single-cell level (Supplementary Fig. S8). Quantitative analyses showed that while MaxComp produced highly heterogeneous compartment assignments, scDIAGRAM aligned more closely with scPCA at both the pooled level and the single-cell level. Since this imaging dataset does not suffer from the sparsity typically encountered in scHi-C data, PCA-based compartment annotation is expected to be stable and informative in this context. Together, these results suggest that the observed discrepancies primarily reflect fundamental differences between interaction-based and distance-based compartment definitions and their sensitivities to single-cell structural variability.

#### scDIAGRAM applied on the human GM12878 cell line

We applied scDIAGRAM to scHi-C data from the human GM12878 cell line at a 1 Mb resolution [13] (Fig. 2f). Compared with scA/B, the pooled scCompartments from scDIAGRAM showed a stronger Pearson correlation with bulk PCA (Correlation = 0.85, compared with scA/B’s correlation of 0.6). Additionally, genomic regions within the same compartment interacted more frequently than those in different compartments, with A−A>A−B and B−B>A−B interactions (Supplementary Fig. S9).

Moreover, we found these scCompartments effectively stratified histone modifications, consistent with observations from bulk PCA. We compared an epigenomic mark enrichment profile with pooled scCompartments (Fig. 2g). The pattern of histone mark enrichment in A/B compartments mirrored the observations from bulk Hi-C [1, 9]. While all histone marks tested were enriched in A compartments, activating marks like H3K36me3 showed a larger enrichment difference between A and B compartments (and a higher Pearson correlation with pseudo-bulk compartments). In contrast, repressive marks such as H3K27me3 and H3K9me3 displayed smaller enrichment differences (and lower correlation with pseudo-bulk compartments), indicating that H3K27me3 and H3K9me3 were more likely to localize to B compartments compared with other marks.

In addition, we evaluated the computational efficiency of scDIAGRAM and compared it with other existing methods. Supplementary Fig. S10 summarizes the runtime and memory usage across approaches. scDIAGRAM exhibits competitive computational efficiency, with runtime comparable to scA/B and lower computational cost than other evaluated methods. These results indicate that scDIAGRAM scales favorably with increasing numbers of cells, supporting its practical applicability to large scHi-C datasets.

### scDIAGRAM applied on the scHi-C data of adult mouse brain cortex

We applied scDIAGRAM to the HiRES dataset from mouse brains [38]. Embedding single cells based on scDIAGRAM-generated compartments revealed major brain cell types, which were similar to those identified using scA/B, scHiCluster, or Higashi (Supplementary Fig. S11A). This suggested that scCompartments alone can effectively distinguish different major cell types.

**Figure 3:**
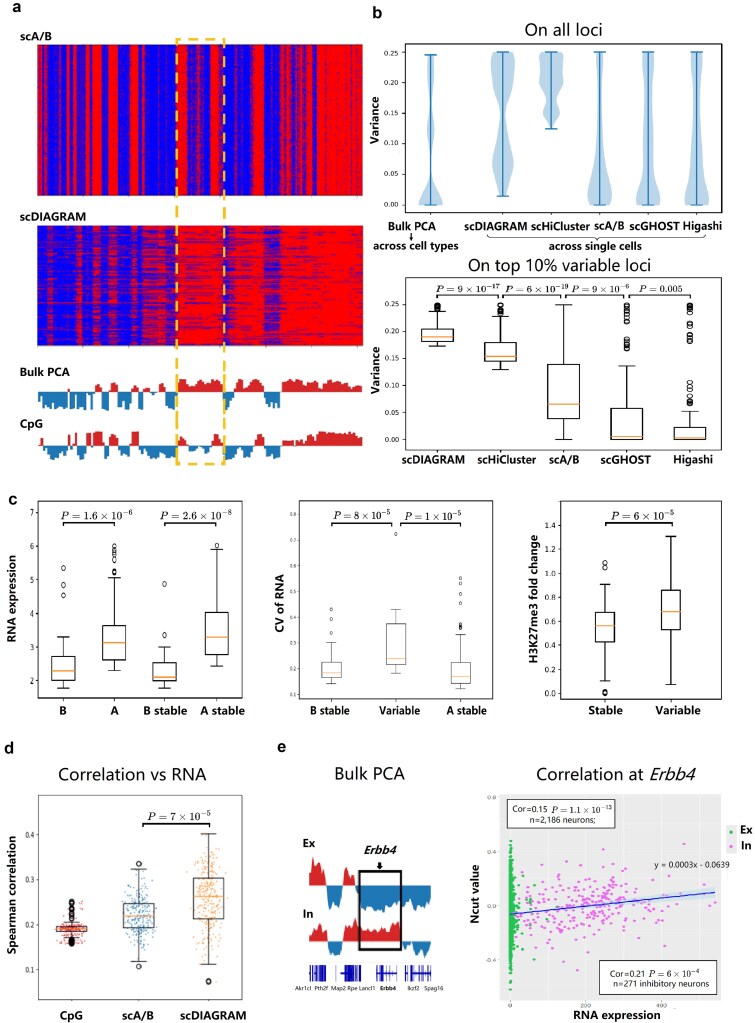
scDIAGRAM applied in a real scHi-C dataset from adult mouse brain cortex. (a) The scCompartments generated by scDIAGRAM is more close to bulk PCA than those generated by scA/B, on the Ex1 dataset from HiRES. Each row represents a cell. The highlighted region is where the CpG density is quite different from bulk PCA (b) scDIAGRAM is more heterogeneous compared with scHiCluster, Higashi, scGHOST, and scA/B. (c) Joint analysis of single-cell transcription and compartments in the same cell type. (d) Spearman correlations with RNA expressions for CpG density, scA/B, and scDIAGRAM, in single cells. (e) Visualization of the bulk PCA at the *Erbb4* locus between excitatory and inhibitory neurons. The RNA expression and real-valued scCompartments (Ncut values from scDIAGRAM) for each single cell, at the *Erbb4* locus. Inhibitory neurons exhibited higher Pearson correlation between RNA and scCompartments than putting inhibitory and excitatory neurons together.

Similar behaviors observed with scDIAGRAM in simulated data were also evident in this real scHi-C dataset. For the Ex1 cell type (chr7, 100 kb), as shown in Fig. 3a, focusing on regions where the CpG signal distinctly differed from bulk PCA, we found that scDIAGRAM was more closely aligned with bulk PCA, whereas scA/B was more reflective of the CpG signal. In Fig. 3b, which considers the entire dataset (chr7, 100 kb), scDIAGRAM displayed greater heterogeneity. We computed the variance of scCompartments across cells for scDIAGRAM, scHiCluster, Higashi, scGHOST, and scA/B, and for bulk PCA, we calculated the variance from bulk compartments across seven cell types. Each violin plot of variances from bulk PCA (across cell types), scDIAGRAM, scA/B, Higashi, and scGHOST exhibited two peaks, indicating stable and variable genomic regions (Fig. 3b). In contrast, scHiCluster showed large variance for all genomic loci, suggesting potential instability during imputation. The bimodal pattern was less pronounced in scA/B, Higashi, and scGHOST, demonstrating that these methods typically generated more homogeneous compartments(also observed in GM12878; Supplementary Fig. S12). Furthermore, when we selected the top 10% most variable loci from real-valued bulk PCA computed across cell types, scDIAGRAM exhibited even greater heterogeneity compared with scHiCluster, scA/B, Higashi, and scGHOST (Fig. 3b). We also compared the pooled scCompartments generated by scDIAGRAM across Ex1 cells with the bulk PCA. The pooled scCompartments always generated better Pearson correlation than a single scCompartment, and scDIAGRAM had better Pearson correlation with bulk PCA than scA/B (Supplementary Fig. S11B).

One limitation of scA/B is that it generates smaller compartments that may not reflect biologically meaningful structures, as compartments typically span multiple megabases. These smaller compartments might correspond to TAD domains or subcompartments, potentially complicating compartment annotations. In contrast, scDIAGRAM produced compartments that were more consistent in size with those from bulk PCA, while both scA/B and scHiCluster tended to generate smaller compartments (Supplementary Fig. S13).

#### Stable and variable single-cell compartments from scDIAGRAM

We applied scDIAGRAM to identify genomic loci with stable and variable scCompartments (Material and methods). Overall, a larger proportion of loci were stable, while variable loci predominantly corresponded to compartmental boundaries and cell-type-specific patterns of genome organization (Supplementary Fig. S14A–C). For each genomic locus, classified as either variable or stable, we calculated the fraction of cells in the A and B compartments. Stable regions were predominantly annotated as A on chromosome 7 and as B on chromosome 1, whereas variable regions exhibited a more balanced distribution between the A and B compartments (Supplementary Fig. S14D and E). These results are consistent with prior researches [35, 38, 45].

In the HiRES dataset, which included both Hi-C and RNA-seq data measured simultaneously for each cell, we were able to correlate the scCompartments identified by scDIAGRAM with RNA expressions, exploring their functional implications. We again focused on the Ex1 cell type at 100 kb resolution. As shown in Fig. 3c, scCompartments associated with higher RNA expression were more active. Stable loci were further categorized into A stable and B stable regions. We found that A stable loci exhibited significantly higher RNA expression compared with B stable loci.

In a manner similar to the analysis of imaging data [44], we examined changes in scCompartments for genes in transcribing (UMIs>10) versus silencing (UMIs<1) states within a single cell type (Ex1, Supplementary Fig. S15A). We found that for nearly 60% of the genes studied, the compartment at their TSS was more active during transcription than when the gene was silenced. Both scDIAGRAM and scA/B produced similar patterns, with scDIAGRAM showing a larger fold change in compartmentalization, suggesting greater heterogeneity (Supplementary Fig. S15A). Regions with variable compartments exhibited greater transcriptional variability (Fig. 3c), with this difference becoming more pronounced when more stringent thresholds were applied (Supplementary Fig. S15B). Here, transcriptional variability was quantified by the coefficient of variation.

Similarly, we also divided genomic loci of GM12878 cells into variable and stable scCompartments and assessed the enrichment of H3K27me3, a histone mark that is enriched in subcompartment B1 (which is unstable) and indicative of facultative heterochromatin at the bulk level [46]. Facultative heterochromatin is a structure that can adopt either open or compact conformations depending on temporal and spatial contexts [9, 46], making it inherently unstable.

We observed significantly higher enrichment of H3K27me3 in variable genomic regions compared with stable regions in the cell population ($P=6\times 10^{-5}$, Fig. 3c). This result is further supported by the known association between H3K27me3-repressed genes and expression heterogeneity [47]. Moreover, we found a moderate Pearson correlation between the variance of scCompartments and the H3K27me3 signal (Pearson Correlation = 0.4, Supplementary Fig. S15C).

#### Comparing scCompartments between two cell types

We compared compartmentalization and transcription between Ex1 and astrocyte (Ast) cell types in the HiRES dataset. Comparing top 100 up- and down-regulated genes in Ex1, we observed elevated pseudo-bulk compartment values for up-regulated genes compared with down-regulated genes (Supplementary Fig. S16A). Conversely, the top 100 compartmental activated genes in Ex1 also exhibited higher transcriptional activity than inactivated genes (Supplementary Fig. S16B). The RNA fold change with all marker genes was correlated with compartmental differences, with Pearson and Spearman correlations typically ranging from 0.15 to 0.3 on different chromosomes (Supplementary Fig. S16C). These correlations were especially pronounced when focusing on the top up- and down-regulated genes/loci (Supplementary Fig. S16D).

For gene markers from all four cell types in the adult mouse brain cortex (identified from RNA), we found that the corresponding scCompartments were more activated in their respective cell types, consistent with findings using scA/B (Supplementary Fig. S17). Moreover, we computed the Spearman correlation between scRNA expression and scCompartments. Compared to scA/B, our method demonstrated higher correlations across most chromosomes (Fig. 3d, Supplementary Fig. S18).

To further validate this connection at single-cell resolution, we used an additional dataset, GAGE-seq on mouse brains [39]. Higher gene expression in a cell often corresponded to a higher real-valued compartment (Supplementary Fig. S18D). For 1913 markers with significantly higher expressions in inhibitory neurons, most showed elevated compartmental values in these neurons compared with excitatory neurons (Supplementary Fig. S18D). Thus, the relationship between compartment and gene expression remained evident at single-cell resolution.

We then illustrated these observations on a specific locus. Following the process in [39], we selected the gene exhibiting the most significant increase in compartmental value (by scDIAGRAM) and RNA expression in inhibitory neurons compared with excitatory neurons. This analysis led us to the same gene *Erbb4* as in [39] when we looked at chr1 (Supplementary Fig. S19A and B).

In pseudo-bulk data, the *Erbb4* locus switched from the stable B compartment (in excitatory neurons) to the stable A compartment (in inhibitory neurons). According to prior studies, the *Erbb4* gene was essential in the central nervous system and has been associated with schizophrenia [48]. As expected, we observed differential A/B compartment values for excitatory and inhibitory neurons, correlated with cell-type-specific expression of the *Erbb4* gene (Fig. 3e). The Pearson correlation of scCompartments and scRNAseq was higher inside inhibitory neurons (Fig. 3e). Additionally, we identified similar compartmental dynamics for the gene *Npas3* on chr12 (Supplementary Fig. S19C and D). This gene is a bHLH transcription factor regulating astrocyte-neuron communication and associated with autism [49]. It displayed a variable B to variable A compartmental switch from excitatory to inhibitory neurons, accompanied by increase in the transcriptional activity. Compared with *Erbb4*, this variable switch showed a lower Pearson correlation between scCompartments and scRNAseq.

### scDIAGRAM applied to the developing mouse embryos

We applied scDIAGRAM to the HiRES dataset during embryogenesis.

#### Compsc-Ncut of Ncut represents compartmental strength

The optimal $F=\frac{1-\lambda _{2}}{2m}$ calculated from the second eigenvalue $\lambda _{2}$ in normalized cut and the total edge weight $m$ of the graph reflects how well the genome is partitioned into two compartments (See Supplementary Methods). We define $-\log _{2}(F)$ as a quantitative measure of compartmental strength, denoted as Compsc-Ncut. Lower Compsc-Ncut values indicate weaker or less distinct compartmental separation.

**Figure 4:**
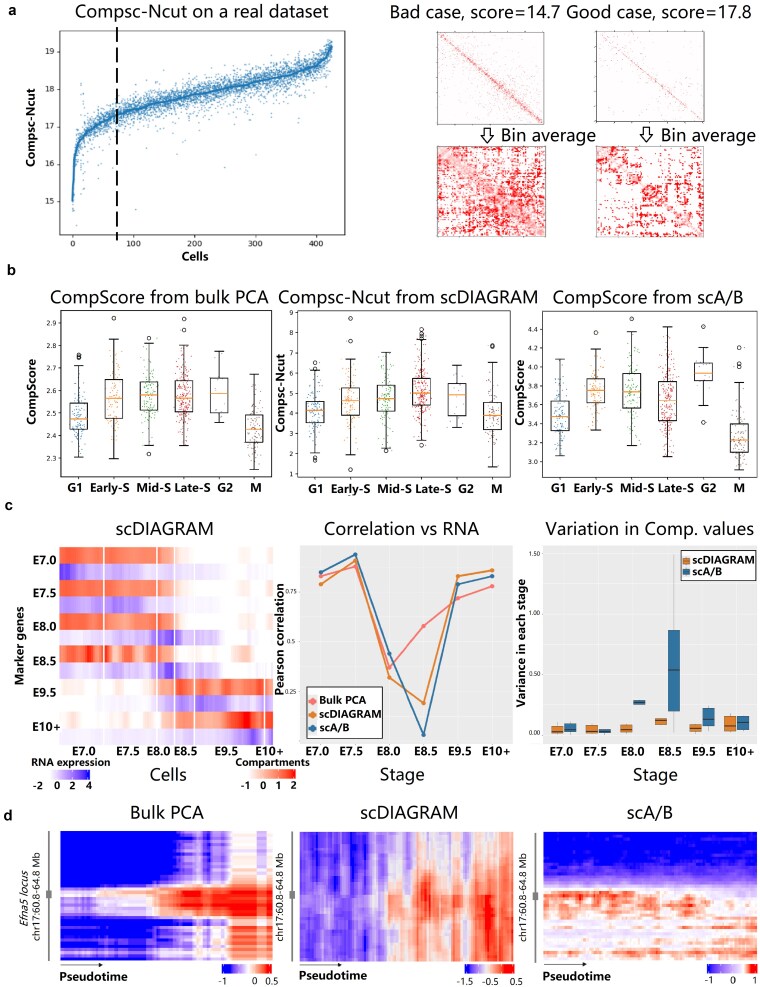
scDIAGRAM applied to the mouse embryonic development. (a) Compsc-Ncut of scDIAGRAM on a real scHi-C dataset and two examples, with larger values of Compsc-Ncut indicating clearer compartmental structure. Each cell was repeated for 10 times and the line was the averaged Compsc-Ncut for each cell. (b) Compartmental strength changes during cell cycle, using the Compsc-Ncut in scDIAGRAM and compartment score [11] for bulk PCA and scA/B. (c) Compartmental values from scDIAGRAM and RNA expressions (left), the Pearson correlation between compartmental values and RNA expressions (middle), and the variance of compartmental values (right) for each set of marker genes at different developmental stages. For each set of marker genes, the compartment annotations are averaged at each metacell. In the heatmap, each row represented a set of marker genes for a specific stage, while each column corresponded to a metacell, ordered according to pseudotime. (d) Compartments of the gene *Efna5* and its neighboring loci at 100 kb resolution. Each column represents a cell, ordered by the pseudotime from left to right. Heatmaps were smoothed by every five neighboring metacells.

We conducted permutation experiments to validate the ability of Compsc-Ncut from Ncut to represent compartmental strength of a Hi-C matrix. We randomly shuffled an Hi-C matrix and mixed it with the original one, breaking the compartmental structure. The Compsc-Ncut decreased as more shuffled matrices were mixed together (Supplementary Methods and Supplementary Fig. S20). In a real scHi-C dataset, we observed a very diversed range of the Compsc-Ncut (Fig. 4a), and we typically used a cutoff value of Compsc-Ncut at 17.3 in practical applications. We also presented two examples to illustrate the effect of Compsc-Ncut, where a lower Compsc-Ncut indicated a weaker compartmental structure (Fig. 4a).

With this tool in hand, we were able to investigate the compartmental variation throughout the cell cycle. In Fig. 4b, we used this Compsc-Ncut as a metric for compartmental strength and tracked its changes throughout the cell cycle. We observed a trend that closely mirrored the acknowledged compartmental change during the cell cycle, which was computed from the compartment score from bulk PCA [11]. In contrast, applying the compartment score to compartments generated by scA/B did not reveal the same trend, with only the maximum and minimum values aligning with what we expected.

#### Dynamic compartmental evolution during development

The HiRES dataset included developing embryos spanning embryonic day 7.0 (E7.0) to day 11.5 (E11.5). single-cell RNA-sequencing (scRNA-seq) analysis revealed two developmental lineages: the neural and mesenchymal lineages, both originating from the epiblast and primitive streak [38]. To mitigate intrinsic noise and cell-cycle effects in the scHi-C data, we constructed “metacells” by aggregating single cells with similar expression profiles followed by trajectory inference and pseudotime analysis (Supplementary Methods). In this study, we specifically focused on the neuronal trajectory to investigate compartmental transition dynamics during development.

Building on similar analyses from [35, 39], we examined compartmental changes associated with stage-specific marker genes. Given the continuous nature of embryogenesis and the potential ambiguity in cell type definitions, we identified marker genes separately for each embryonic stage. For each stage, all other stages were treated as controls, and differential expression analysis was performed using the Seurat package (see Supplementary Methods). Subsequent analyses focused on the top 100 marker genes at each stage, ordered by the fold change.

In Fig. 4c, we visualized compartmental transitions of these marker genes during development, averaging the compartment values within each marker gene set. We assigned compartments for each metacell using bulk PCA, scDIAGRAM, and scA/B. For bulk PCA, compartments were assigned based on pseudo-bulk matrices constructed from cells at each developmental stage.

Compartmental changes exhibited a strong Pearson correlation with RNA expression for marker genes at the early (E7.0 and E7.5) and late (E9.5 and E10+) stages of development (Supplementary Fig. S21). In contrast, markers at intermediate stages (E8.0–E8.5) showed markedly reduced correlations (Fig. 4c, middle). Marker genes specific to particular developmental stages generally displayed more active scCompartments, except at E8.5, where all methods produced highly variable scCompartments with only slight enrichment (Fig. 4c, right). These observations are consistent with the known intermixing of ectodermal and mesodermal cells prior to E8.5, which begin to diverge only afterward [38], further highlighting the cellular heterogeneity at this stage. Additionally, across developmental stages, scDIAGRAM showed better alignment with bulk PCA than scA/B (Supplementary Fig. S21).

A closer examination of stage E8.0 revealed notable differences between scDIAGRAM and scA/B. We identified the gene Efna5 that showed opposing compartmental transitions when comparing bulk PCA and scA/B (Fig. 4d). In contrast to scA/B, scDIAGRAM aligned with bulk PCA, displaying an elevated compartmental signal, while scA/B indicated a more inactive state at this locus. Efna5 encodes an axon guidance protein involved in late-stage nervous system development by preventing axon bundling [50]. As shown in Fig. 4d, the compartment patterns of neighboring loci also more closely resembled those from bulk PCA when inferred by scDIAGRAM. scRNA-seq analysis further revealed that Efna5 expression increased at early developmental stages and declined thereafter, potentially explaining the discordance between expression and the compartment patterns detected by scA/B, as previously noted in [51].

### scDIAGRAM applied to the acute myeloid leukemia

We applied scDIAGRAM to the HiRES dataset of human AML. Previous bulk analyses had identified subtype-specific A/B compartment patterns in AML [52]. Consistent with this, scRNA-seq embeddings revealed patient-specific clustering (Supplementary Fig. S22). We therefore focused our downstream analyses on compartmental differences across patients.

**Figure 5:**
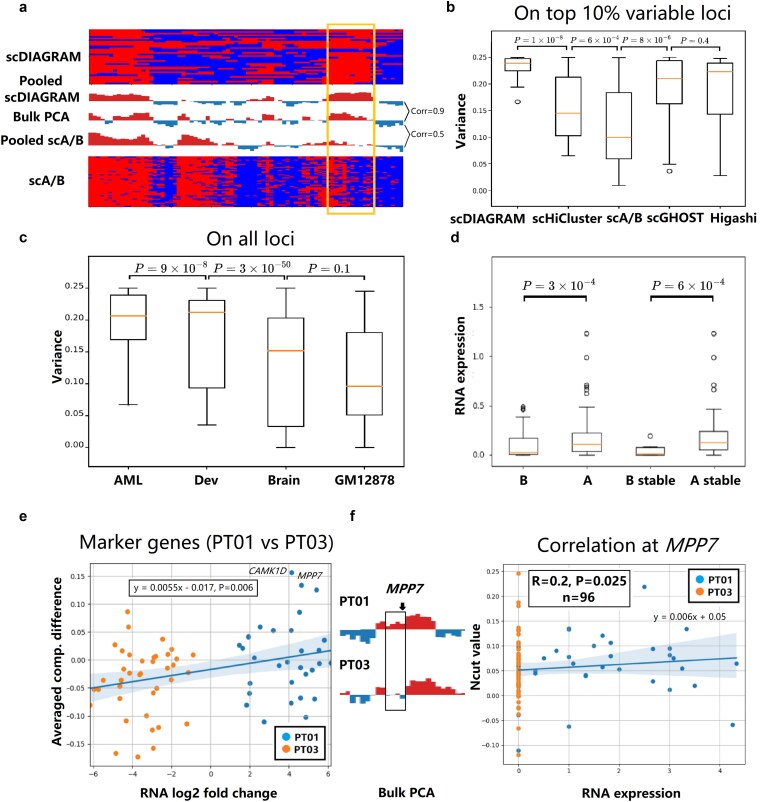
scDIAGRAM applied to the AML. (a) scCompartments inferred by scDIAGRAM showed closer agreement with bulk PCA than those from scA/B in the HiRES dataset of patient PT03. Each row represented a single cell. The highlighted region marked a locus where scA/B substantially deviated from bulk PCA. (b) scDIAGRAM captured greater compartmental heterogeneity compared to scHiCluster, Higashi, scGHOST, and scA/B. (c) Compartmental variance of scDIAGRAM was evaluated across different datasets. (d) Average RNA expression levels were compared between loci assigned to A and B compartments, as well as among stable A/B loci. (e) Changes in compartmentalization were correlated with gene expression differences for marker genes between patients PT01 and PT03. (f) Bulk PCA signals at the MPP7 locus were visualized for PT01 and PT03, along with corresponding RNA expression and scDIAGRAM Ncut values at the single-cell level.

For chromosome 10 in Patient 03 (PT03) (Fig. 5a), scDIAGRAM demonstrated stronger concordance with bulk PCA than scA/B, as indicated by a higher Pearson correlation. Notably, in a region where scA/B showed substantial deviation from the bulk reference, scDIAGRAM remained closely aligned. In Fig. 5b (chromosome 10 across all patients), scDIAGRAM also exhibited greater compartmental heterogeneity compared with other methods. Following the approach in Fig. 3b, we computed the variance of scCompartments across cells. At the top 10$\%$ most variable loci—defined by bulk PCA computed across cell types—scDIAGRAM consistently showed higher variability than scHiCluster, scA/B, Higashi, and scGHOST across all patients.

Furthermore, cross-dataset comparisons using scDIAGRAM (Fig. 5c) revealed that the datasets of AML and developing embryos exhibited significantly higher scCompartment variability than both brain cells and the GM12878 cell line. This pattern is consistent with biological intuition: cancer cells and developing cells are expected to exhibit greater heterogeneity in chromatin organization due to their dynamic regulatory states, whereas terminally differentiated or steady-state cells tend to be more stable and homogeneous.

We next examined the relationship between compartments and transcriptional activity. As shown in Fig. 5d, loci annotated as A compartments were generally more transcriptionally active than B compartments. Moreover, stable A/B loci exhibited greater differences in gene expression, highlighting the functional relevance of compartmental identity. In Fig. 5e, comparing patients PT01 and PT03, the RNA fold change of all marker genes $(n=70$, adjusted $P<.05)$ was significantly correlated with the corresponding average compartmental differences (Spearman’s $r=0.32$, $P=.003$). This association was consistently observed across other patient comparisons as well (Supplementary Fig. S22).

Finally, in Fig. 5f, using the same approach as in Fig. 3e, we identified the gene MPP7, which exhibited the most pronounced increase in both compartmental value and RNA expression in PT01 compared with PT03 (Supplementary Fig. S22). Bulk PCA analysis confirmed a B-to-A compartment switch at this locus between the two patients. This shift was mirrored by changes in MPP7 expression and scDIAGRAM-inferred compartment values, suggesting a coordinated regulatory transition. While MPP7 has been previously implicated in tumorigenesis, including in breast cancer [53], our findings suggest it may also play a role in AML, offering a novel biological prediction. A recent pan-cancer analysis based on public cancer databases has examined MPP7 across various cancer types, including AML [54], supporting the potential relevance of this gene in leukemogenesis. In addition to MPP7, we also identified known AML-associated genes, such as CAMK1D [55], that showed similar RNA and compartmental differences between PT01 and PT03 (Supplementary Fig. S22).

## Conclusion

In this study, we introduced scDIAGRAM, a computational method designed to detect single-cell chromatin compartments directly from scHi-C data using statistical modeling and graph partitioning. The core compartment inference step relies solely on Hi-C interaction information and does not incorporate external signals or imputation, allowing compartments to be identified independently for each scHi-C matrix. To assign the conventional A/B labels to the inferred compartments, scDIAGRAM uses CpG density as a post hoc labeling reference, following common practice in A/B compartment analysis. This separation between compartment detection and label assignment enables scDIAGRAM to preserve intrinsic cell-to-cell heterogeneity while maintaining interpretability within the standard A/B framework. As a result, scDIAGRAM enables us to capture the inherent heterogeneity of compartments within an scHi-C dataset, providing a more accurate representation of chromatin organization across diverse cell types. Through extensive simulations and real data analysis, we demonstrated that scDIAGRAM effectively annotated compartments across various cell types. We also identified cell-type-specific compartments and explored their association with gene expression profiles in the HiRES dataset.

The differences in performance across methods for compartment annotation can be attributed to their distinct modeling assumptions and data-processing strategies. scA/B incorporates CpG density information during compartment assignment; as a result, under very low sampling rates, its annotations tend to be increasingly influenced by CpG density rather than interaction-derived signals from sparse Hi-C data. Both scHiCluster and Higashi rely on imputation to enhance data quality. While this strategy can effectively reduce noise, it may also smooth cell-specific variability. This effect is particularly pronounced for Higashi, which further integrates information across neighboring cells during imputation, potentially attenuating heterogeneity at the single-cell level. Hickit infers three-dimensional chromatin structures from scHi-C data and was originally designed and evaluated on relatively high-coverage datasets. Under extreme sparsity, reconstructing reliable 3D structures becomes challenging that may partly explain its reduced performance at very low sampling rates.

Beyond differences in modeling assumptions and data-processing strategies, existing methods also differ substantially in terms of interpretability. Methods such as Higashi [21] and scGHOST [35] have demonstrated the potential of artificial intelligence and deep neural networks (DNNs) for annotating compartments and subcompartments within scHi-C datasets. While DNN-based approaches can achieve strong performance by leveraging shared information across cells and large training datasets, their reliance on complex architectures often makes it challenging to interpret how compartment annotations are derived at the single-cell level. In contrast, scDIAGRAM functions as a plug-and-play method that directly analyzes individual scHi-C matrices without the need for extensive training data or cross-cell information sharing. This design makes scDIAGRAM particularly well suited for studying compartmental heterogeneity and gene regulatory relationships, while avoiding the interpretability and scalability challenges commonly associated with DNN-based methods.

The relationship between compartments, epigenomic features, and transcription is highly complex. Further quantitative analyses are needed to fully uncover these connections, and integrating epigenetic data with Hi-C could help improve the accuracy of compartment annotations. Additionally, understanding the mechanisms driving cell-to-cell compartmental variability is a significant challenge. This variability may stem from a range of factors, including cell type, cellular states, biological processes (such as the cell cycle), intrinsic cellular dynamics, and technical biases. Future research should focus on disentangling these factors and minimizing technical biases in scDIAGRAM, ultimately enhancing its robustness.

The assumption of constant interaction parameters within each block between adjacent CPs represents a deliberate modeling simplification. This assumption was introduced to facilitate reliable CP detection, rather than to capture fine-scale interaction variability within compartments. Empirically, we found that this simplification had a limited impact on final compartment inference. One possible explanation is that when the number of CPs is sufficiently large, each block spans a relatively small genomic interval, making locally constant parameters a reasonable approximation. From this perspective, the current formulation strikes a practical balance between model simplicity and robustness in the context of sparse scHi-C data. Future developments could further refine the underlying probabilistic framework. For example, incorporating explicit distance-dependent effects or additional technical covariates (such as GC content and mappability) may provide a natural extension of scDIAGRAM and further improve the accuracy of inferred A/B compartments.

**Key Points**

- We introduce scDIAGRAM (single-cell compartments annotation by DIrect stAtistical modeling and GRAph coMmunity detection), a novel statistical framework for annotating A/B chromatin compartments from individual sparse single-cell Hi-C data. Unlike existing tools, scDIAGRAM detects compartments without imputation or external genomic features, while external information (e.g. CpG density) is used only for the final A/B labeling. The method preserves cell-to-cell heterogeneity and enables robust analysis across diverse biological contexts.
- scDIAGRAM first formulates compartment annotation as a 2D change-point detection problem, employing a Bayesian Markov Chain Monte Carlo algorithm to identify potential compartment boundaries. It then constructs a graph from the resulting segmentation and applies graph partitioning to divide the genome into two major compartment groups, effectively capturing spatial chromatin organization at single-cell resolution.
- We carry out a series of comparison experiments. scDIAGRAM demonstrated outstanding performance on simulated data with ground truth, and showed strong concordance with single-cell RNA-sequencing and epigenomic profiles in real datasets. It successfully captured known variation during embryonic development and revealed subtype-specific patterns in acute myeloid leukemia, highlighting both accuracy and biological relevance.

## Supplementary Material

scDIAGRAM_SI_tot2_clean_bbag096

## Data Availability

The single-cell AML data generated in this study have been deposited in Gene Expression Omnibus (GEO) under accession code GSE302267. AML subtypes were provided in Supplementary Table S1. The corresponding processed cell-by-gene scRNA-seq data have also been uploaded to the GEO. Other data are publicly available, and source code of scDIAGRAM are on Github (https://github.com/Ge-lab-pku/scDIAGRAM) and Zenodo (https://doi.org/10.5281/zenodo.15855256).
