# Supplementary material for: scDIAGRAM: detecting chromatin compartments from individual single-cell Hi-C matrix without imputation or reference features
Source: Brief Bioinform. 2026 Mar 8;27(2):bbag096. doi: 10.1093/bib/bbag096 (PMC12967335; doi:10.1093/bib/bbag096)
Supplement: scDIAGRAM_SI_tot2_clean_bbag096 [file scdiagram_si_tot2_clean_bbag096.pdf]

scDIAGRAM: Detecting Chromatin Compartments from Individual Single-Cell Hi-C Matrix without Imputation or Reference Features

Yongli Peng, Yujing Deng, Menghan Liu, Zhiyuan Liu,
Ya-Hui Li, Xiang-Yu Zhao, Dong Xing†, Jinzhu Jia† and Hao Ge†

†To whom correspondence should be addressed. Email: haoge@pku.edu.cn;jzjia@math.pku.edu.cn;dxing@pku.edu.cn

1 Supplementary Methods

1.1 The derivation and details of normalized cut

The objective function can be expressed into a quadratic form:

$$\frac{R}{\kappa_1\kappa_2} = \frac{2m - \mathbf{s}^T\mathbf{A}\mathbf{s}}{(2m)^2}, \; s_i = \begin{cases} \sqrt{\kappa_2/\kappa_1} & \text{if} \quad i \text{ is in compartment } 1, \\ -\sqrt{\kappa_1/\kappa_2} & \text{if} \quad i \text{ is in compartment } 2, \end{cases}$$

where $\mathbf{A}$ is the adjacency matrix and m represents the total weight of all edges in the graph.

Next, we relaxed the above discrete $\mathbf{s}$ into a vector in $\mathbb{R}^n$, transforming the problem into:

$$\max_{\mathbf{s}\in\mathbb{R}^n} \mathbf{s}^T\mathbf{A}\mathbf{s}, \text{ s.t. } \sum_i k_i s_i = 0, \sum_i k_i s_i^2 = 2m,$$

where k_i is the degree of node i.

This problem can be solved by introducing Lagrange multipliers λ, μ for the two constraints and differentiating, i.e.

$$\mathbf{A}\mathbf{s} = \lambda\mathbf{D}\mathbf{s} + \mu\mathbf{k},$$

where $\mathbf{k}$ is the vector with elements k_i and $\mathbf{D}$ is the diagonal matrix with elements $D_{ii} = k_i$. Multiplying on the left by $\mathbf{1}^T$ and using $\mathbf{1}^T\mathbf{A} = \mathbf{1}^T\mathbf{D} = \mathbf{k}^T$, $\mathbf{k}^T\mathbf{s} = 0$ one got $\mu = 0$. Hence the solution should satisfy the generalized eigenvector equation

$$\mathbf{A}\mathbf{s} = \lambda\mathbf{D}\mathbf{s}.$$

This equation can be explicitly solved by considering the eigen decomposition of $\mathbf{D}^{-1/2}\mathbf{A}\mathbf{D}^{-1/2}$. We denoted the solutions to this generalized eigenvector equation were $(\nu_1, \nu_2, \cdots, \nu_{K+1})$ with the corresponding eigenvalues $(\lambda_1 \geq \lambda_2 \geq \cdots \geq \lambda_{K+1})$. The following properties hold:

- each $\lambda_i \leq 1$, $\lambda_1 = 1$ and $\nu_1 = \mathbf{1} = (1, 1, 1\cdots)^T$;
- $\frac{R}{\kappa_1\kappa_2} = \frac{2m-\mathbf{s}^T\mathbf{A}\mathbf{s}}{(2m)^2} = \frac{1-\lambda_i}{2m}$ if $\mathbf{s} = \nu_i$ and $\sum_i k_i s_i^2 = \mathbf{s}^T\mathbf{D}\mathbf{s} = 2m$, for any i.

Since $\nu_1 = \mathbf{1}$ can not satisfy the constraint $\mathbf{k}^T\mathbf{s} = 0$, we turned to ν_2, which is exactly the solution of the optimization problem.

To obtain a discrete $\mathbf{s}$ for graph partitioning, $\mathbf{s}$ was rounded, typically using 0 as a threshold and nodes were assigned to the two compartments based on the sign of $\mathbf{s}$ (according to Newman (2013), the result was robust to the rounding strategy chosen for division). The compartmental value of each locus just inherited from the value of the corresponding node, i.e. the group of loci separated by CPs.

1.2 MCMC algorithm for the posterior distribution

The pseudocode was provided in Algorithm 1.

To be specific, we used $v_i = 1$ to denote the presence of a CP between the i-th and $(i+1)$-th loci, and $v_i = 0$ otherwise. Starting from the current state $(v_i)_{1\leq i\leq n-1}$, we randomly selected $j \in I_1 = \{i : v_i = 1\}$, $k \in I_0 = \{i : v_i = 0\}$ and proposed the next candidate state $(v_i')_{1\leq i\leq n-1}$ which moves one CP at position j to position k. Each pair (j, k) had equal probability $\frac{1}{2K(n-1-K)}$ with $|I_1| = K$. This proposal was symmetric.

Algorithm 1: Metropolis-Hasting Sampling

Input: Matrix $\{Y_{ij}\}$, number of iterations N, number of changing points K;

Initialize the positions of all CPs $(v_i^{(0)})$ (randomly choose K positions as CPs), $t = 0$;

Output: $(v_i^{(1)}), \cdots, (v_i^{(N)})$;

for $t = 0, 1, \ldots, N-1$ **do**

  Randomly select j from $I_1 = \{i : v_i^{(t)} = 1\}$ and k from $I_0 = \{i : v_i^{(t)} = 0\}$;

  define $v_i' = \begin{cases} 1 - v_i^{(t)}, & \text{if } i = j \text{ or } k \\ v_i^{(t)}, & \text{otherwise} \end{cases}$

  $p_{acc} = \alpha(v_1^{(t)}, \cdots, v_{n-1}^{(t)}; v_1', \cdots, v_{n-1}')$ # α defined in Appendix;

  if $u \sim U(0,1) \leq p_{acc}$ **then**

    Accept candidate $(v_i^{(t+1)}) \leftarrow (v_i')$

  else

    Reject candidate $(v_i^{(t+1)}) \leftarrow (v_i^{(t)})$

  end

end

We accepted this candidate $(v_i')_{1\leq i\leq n-1}$ with probability $\alpha(v_1, \cdots, v_{n-1}; v_1', \cdots, v_{n-1}')$ (defined in Supplementary Methods 1.3), which was derived from the likelihood ratio between the two states. In this way, the sampling sequence formed a reversible Markov chain and the distribution converged to the posterior distribution in the limit.

Since MCMC involves inherent randomness, we repeated the process multiple times (typically 5–10 repetitions) to improve robustness.

Finally, we selected the state with the maximal likelihood value among all the sampled states as an approximation to the MLE solution.

1.3 The acceptance probability in MCMC

Specifically, given the present state $(v_i)_{1\leq i\leq n-1}$, we randomly moved one CP at the position j to an unoccupied position k, proposing a candidate state.

We accepted this proposal $(v_i')_{1\leq i\leq n-1}$ with probability $\alpha(v_1, \cdots, v_{n-1}; v_1', \cdots, v_{n-1}')$, which was the minimum of the likelihood ratio between these two states and 1.

The likelihood was just the posterior (defined in Methods of the main text), so

$$\begin{aligned}&\alpha(v_1, \cdots, v_{n-1}; v_1', \cdots, v_{n-1}') \\ = \quad &\min\left\{1, \frac{\prod_{l\neq k,k+1} A(v_i, k, l)}{\prod_{l\neq j,j+1} A(v_i, j, l)} \times \frac{B(v_i, k)}{B(v_i, j)}\right\}, \end{aligned} \tag{1}$$

in which

$$\begin{aligned}A(v_i, k, l) = &\frac{\hat{r}_{kl}^{S_{kl}} \hat{r}_{k+1,l}^{S_{k+1,l}}}{(\alpha_{kl}\hat{r}_{kl} + (1-\alpha_{kl})\hat{r}_{k+1,l})^{S_{kl}+S_{k+1,l}}} \\ \times &\frac{(1-\hat{r}_{kl})^{N_{kl}-S_{kl}}(1-\hat{r}_{k+1,l})^{N_{k+1,l}-S_{k+1,l}}}{[\alpha_{kl}(1-\hat{r}_{kl}) + (1-\alpha_{kl})(1-\hat{r}_{k+1,l})]^{N_{kl}+N_{k+1,l}-S_{kl}-S_{k+1,l}}} \\ \times &\frac{\exp\left(-\frac{1}{2\hat{\sigma}^2}(S_{kl}m_{kl}^2 + S_{k+1,l}m_{k+1,l}^2)\right)}{\exp\left(-\frac{1}{2\hat{\sigma}^2}(S_{kl}+S_{k+1,l})(\beta_{kl}m_{kl} + (1-\beta_{kl})m_{k+1,l})^2\right)},\end{aligned}$$

where $\alpha_{kl} = \frac{N_{kl}}{N_{kl}+N_{k+1,l}}$, $\beta_{kl} = \frac{S_{kl}}{S_{kl}+S_{k+1,l}}$ and m_{kl} was the sample mean of nonzero entries in the (k,l)-block. Defined as before, N_{kl} was the sample size of the (k,l)-block and $S_{kl} = \sum_{i\in g_k, j\in g_l} 1_{\{X_{ij}\neq 0\}}$ was the nonzero sample size in the (k,l)-block.

The expression of $B(v_i, k)$ is

$$\begin{aligned}
B(v_i,k) &= \left(\frac{\hat{r}_{kk}}{\overline{r}_k}\right)^{S_{kk}} \left(\frac{1-\hat{r}_{kk}}{1-\overline{r}_k}\right)^{N_{kk}-S_{kk}} \\
&\times \left(\frac{\hat{r}_{k+1,k+1}}{\overline{r}_k}\right)^{S_{k+1,k+1}} \left(\frac{1-\hat{r}_{k+1,k+1}}{1-\overline{r}_k}\right)^{N_{k+1,k+1}-S_{k+1,k+1}} \\
&\times \left(\frac{\hat{r}_{kk}}{\overline{r}_k}\right)^{2S_{k,k+1}} \left(\frac{1-\hat{r}_{k,k+1}}{1-\overline{r}_k}\right)^{2N_{k,k+1}-2S_{k,k+1}} \\
&\times \frac{\exp\left(-\frac{1}{2\hat{\sigma}^2}(S_{kk}m_{kk}^2 + S_{k+1,k+1}m_{k+1,k+1}^2 + 2S_{k,k+1}m_{k,k+1}^2)\right)}{\exp\left(-\frac{1}{2\hat{\sigma}^2}(S_{kk} + S_{k+1,k+1} + 2S_{k,k+1})\overline{m}_k^2\right)},
\end{aligned}$$

where $\overline{r}_k = A_k\hat{r}_{kk} + B_k\hat{r}_{k+1,k+1} + 2C_k\hat{r}_{k,k+1}$ was the weighted average of $(\hat{r}_{kk}, \hat{r}_{k,k+1}, \hat{r}_{k+1,k+1})$, and $\overline{m}_k = D_k m_{kk} + E_k m_{k+1,k+1} + 2F_k m_{k,k+1}$ was the weighted average of $(m_{kk}, m_{k,k+1}, m_{k+1,k+1})$.

The weights $A_k, B_k, C_k, D_k, E_k, F_k$ were given by

$$\begin{aligned}
A_k &= \frac{N_{k,k}}{N_{k,k} + N_{k+1,k+1} + 2N_{k,k+1}}; \\
B_k &= \frac{N_{k+1,k+1}}{N_{k,k} + N_{k+1,k+1} + 2N_{k,k+1}}; \\
C_k &= \frac{N_{k,k+1}}{N_{k,k} + N_{k+1,k+1} + 2N_{k,k+1}}; \\
D_k &= \frac{S_{k,k}}{S_{k,k} + S_{k+1,k+1} + 2S_{k,k+1}}; \\
E_k &= \frac{S_{k+1,k+1}}{S_{k,k} + S_{k+1,k+1} + 2S_{k,k+1}}; \\
F_k &= \frac{S_{k,k+1}}{S_{k,k} + S_{k+1,k+1} + 2S_{k,k+1}}.
\end{aligned}$$

1.4 The Gelman-Rubin statistics for MCMC convergence

The Gelman-Rubin statistic was used to assess MCMC convergence by comparing the within-chain and between-chain variances of multiple, independent MCMC chains.

The statistic required m chains, each with n steps. It first computed the between-chain variance B and the within-chain variance W:

$$B = \frac{n}{m-1}\sum_{j=1}^{m}\left(\bar{\theta}_{.j} - \bar{\theta}_{..}\right)^2, W = \frac{1}{m}\sum_{j=1}^{m} s_j^2,$$

where $\bar{\theta}_{.j}$ was the mean of chain j and $\bar{\theta}_{..}$ is the overall mean across all chains and s_j^2 was the variance of the chain j. These quantities estimate the target posterior variance $\widehat{\text{Var}}(\theta \mid \text{data}) = \frac{n-1}{n}W + \frac{1}{n}B$. The Gelman-Rubin statistic was then defined as $\hat{R} = \sqrt{\frac{\widehat{\text{Var}}(\theta| \text{ data })}{W}}$.

When the MCMC converged, the between-chain variance B shall be close to 0 and hence $\hat{R}$ was close to 1. $\hat{R} < 1.05$ is used as a typical threshold for indicate approximate convergence.

1.5 Quality control of compartmental structure using scDIAGRAM

In some cells, the compartmental structure appeared ambiguous, making it necessary to exclude them from downstream analyses. We computed Compsc-Ncut for each cell and applied a cutoff (typically 17.0–17.3) to filter out cells with poorly defined compartment patterns.

We also used the Pearson correlation between the CpG density and the annotation generated by scDIAGRAM as an additional filtering criterion. A lower correlation value indicated a significant deviation from expected patterns. A threshold between 0.1 and 0.2 was typically sufficient to identify and exclude such cases.

1.6 Compartmental strength and normalized cut

We compared the relation between the Compsc-Ncut and the original CompScore defined in Nagano et al. (2017). The original CompScore was:

$$\text{CompScore} = \log_2\left(\frac{2\,(O_{AA}+O_{AB})\,(O_{BB}+O_{AB}))}{mO_{AB}}\right),$$

where O_{AA}, O_{AB}, O_{BB} were total contact numbers between A-A, A-B and B-B compartments, m was the total contacts in the scHi-C matrix (the edge number in the graph). From Newman (2013) we defined the Compsc-Ncut as:

$$\text{Compsc-Ncut} = -\log_2(F),$$
$$F = \frac{O_{AB}}{\kappa_A\kappa_B} = \frac{O_{AB}}{(O_{AA}+O_{AB})\,(O_{BB}+O_{AB})},$$

where κ_A, κ_B were the total contact number (the total degrees) in A/B compartment.
Therefore we can derive CompScore $-$ Compsc-Ncut $= \log_2(2/m)$. When comparing compartment strength in real scHi-C matrices or balanced bulk Hi-C matrices, if the total contacts didn't change much during the comparison, these two metrics were equivalent.

We implemented some permutation experiments to validate the relation between Compsc-Ncut and the compartmental strength. These permutations would disrupt the compartmental structure.

First, we performed random shuffling (reordering) of the genomic bins in the Hi-C matrix. Shuffling did not affect the graphical structure and the compartments were unchanged, but the binary division changed. To disrupt the compartmental structure, we then mixed the original data with the shuffled data (just adding them together). By adjusting the weight of the shuffled data upon mixing, we can also control the extent to which the compartmental structure was disrupted.

1.7 Simulated scHi-C datasets

Downsampling of pseudo-bulk Hi-C data

To generate simulated scHi-C datasets, we performed downsampling on pseudo-bulk Hi-C data derived from the HiRES Liu et al. (2023) dataset, i.e. the excitatory neuron cluster 1 (Ex1) and mixed late mesenchyme (MLM) cell types at 100 kb resolution. The results from bulk PCA served as the ground truth of compartmentalization.

At a given downsampling rate 1/r, we randomly selected 1/r of all the sequencing reads in the pseudo-bulk matrix to create a sampled contact map. This approach was equivalent to applying binomial sampling. The downsampling procedure was repeated 50 times at each rate. The choice of downsampling rates was guided by the contact numbers observed in the pseudo-bulk data and real single-cell data. The rates used in this study were 1/400, 1/800, 1/1200 (close to real scHi-C dataset), 1/1600, 1/2400 and 1/3200.

Downsampling from single-cell 3D genome imaging data

We further simulated an additional scHi-C dataset using a recent 3D genome imaging dataset comprising 3,029 chromosomes (chr2) of single cells from Su et al. (2020). This dataset provided chromosome labeling at 250 kb resolution. We generated synthetic scHi-C matrices at 1 Mb resolution from this data, just like the processing steps in Zhang et al. (2022).

First we averaged the coordinates of every four 250 kb segments to derive the spatial coordinates of each 1 Mb segment. As demonstrated in Bintu et al. (2018), the inverse of the spatial distance was strongly correlated with Hi-C contact frequencies between pairs of genomic loci. Using this relationship, we constructed the original dataset by taking the inverse of the distance matrix (the diagonal all set to zero in the original data). The PCA of the original matrix was used as the ground truth for subsequent analysis.

Reads were then randomly sampled with probabilities proportional to the values in this ground-truth matrix. The law of large numbers ensured that the sampled contact matrice converged to the ground truth as the total contact number n increased. We generated samples with contact numbers of 250, 500, 1,000, 2,500, 5,000, and 10,000. Supplementary Figure S6 listed the contact numbers per cell for various

scHi-C datasets on the same chromosome. A typical scHi-C dataset had median contact number ranging from 500 to 5,000 while recent high coverage datasets had median contact number larger than 10,000.

Downsampling of high coverage scHi-C data

We further assessed the robustness of scDIAGRAM to sequencing depth by downsampling a high-coverage scHi-C dataset. We used the Dip-C dataset, which includes data from 14 GM12878 cells (chr1) at 500 kb resolution Tan et al. (2018). The corresponding 3D structural models were obtained using the Hickit package. Similar to the above approach for downsampling single-cell 3D imaging data, we took the inverse of the distance matrix and applied PCA to obtain the ground truth for this analysis. Following Zhang et al. (2022), 500 kb were chosen here so that Hickit can produce 3D structures at full coverage, as 3D structures at higher resolutions would skip bins with insufficient Hi-C contacts.

Next, we downsampled the Dip-C dataset to 50%, 25%, 10%, 5%, and 1% of the original read coverage, following the same procedure used for downsampling pseudo-bulk Hi-C data. The 1% read coverage corresponds to the median number of contacts per cell typically observed in scHi-C datasets (Supplementary Figure S6).

Comparing with MaxComp

The recently published MaxComp Zhan et al. (2024) leverages three-dimensional spatial chromosome structures to annotate A/B compartments based on geometric considerations. Specifically, MaxComp represents each 3D chromosome structure as an undirected, weighted graph and reformulates compartment annotation as a Max-cut problem, which is solved using semidefinite programming (SDP) to partition the graph into two structural compartments.

We compared MaxComp with scDIAGRAM using the same single-cell 3D genome imaging dataset (DNA MERFISH data from human IMR90 cells) reported in Su et al. (2020), at 3 Mb resolution on chromosome 1. In MaxComp, the graph weights were constructed as

$$w_{ij} = \frac{|z_i - z_j| \, \|\boldsymbol{x}_i - \boldsymbol{x}_j\|_2}{(|g_i - g_j|)^{1/2}},$$

where $\boldsymbol{x}_i$ and $\boldsymbol{x}_j$ denote the 3D coordinates of loci i and j, g_i and g_j denote their genomic positions, and z_i and z_j denote the z-scores of the measured distances between each locus and the closest nuclear speckle. In this dataset, speckle distance information was provided in Su et al. (2020), which we directly used to compute the MaxComp graph.

For PCA and scDIAGRAM, we constructed a proxy Hi-C contact matrix from the same imaging data by using the inverse of the spatial distance,

$$f_{ij} = \frac{1}{\|\boldsymbol{x}_i - \boldsymbol{x}_j\|_2},$$

so that these interaction-based methods could be applied in a consistent manner. We observed that the two matrices, w_{ij} and f_{ij}, were negatively correlated (Pearson's correlation $r \approx -0.4$).

Simulating Hi-C data via FreeHi-C

We simulated Hi-C data using FreeHi-C Zheng and Keleş (2020), based on bulk Hi-C data from the malaria parasite *Plasmodium falciparum* 3D7. All default parameters provided by the authors were used, except for the sequencing depth, which was varied to assess robustness. Specifically, sequencing depths of 6×10^5, 1.2×10^6, 2.4×10^6, 4.8×10^6, 7.2×10^6, and 9.6×10^6 were considered.

After simulation, we applied scDIAGRAM, PCA, and scA/B to chromosome 14 (the longest chromosome) at 50 kb resolution. Compartment annotations derived from PCA on the original bulk Hi-C data were used as the ground truth. For each sequencing depth, the simulation and evaluation procedure was repeated 10 times.

1.8 Preprocessing of real scHi-C and scRNA-seq data

The mouse reference genome (GRCm38) and gene annotations (ALL) were downloaded from the GENCODE M23 release. The human reference genome (GRCh37 for GM12878 and GRCh38 for AML) and gene annotations were downloaded from UCSC Genome Browser Kent W.J and et al (2002). The CpG density data were computed from these reference genomes.

We used cooler Abdennur and Mirny (2020) and cooltools Open2C et al. (2024) to apply band normalization, matrix balancing, and compartment annotation to our pseudo-bulk Hi-C matrices. Single-cell Hi-C matrices also underwent band normalization prior to bin averaging. However, the extreme sparsity of raw scHi-C data makes it impossible to perform reliable band normalization or matrix balancing using only individual single-cell matrices. To overcome this, we recommend borrowing the expected contact frequency vectors and balancing weights calculated from the pseudo-bulk matrices when normalizing and balancing each single-cell dataset.

The HiRES dataset from Liu et al. (2023) was downloaded from the GEO (GSE223917). The data processing method for AML in this study follows the same procedure as in Liu et al. (2023). The GAGE-seq dataset was downloaded from the GEO (GSE238001). The imaging dataset Su et al. (2020) was obtained from Zenodo (https://doi.org/10.5281/zenodo.3928890). The Dip-C high coverage scHi-C dataset Tan et al. (2018) was downloaded from the GEO (GSE117876). The processed scHi-C data of the GM12878 cell line were downloaded from Kim et al. (2020) (https://noble.gs.washington.edu/proj/sc hic-topic-model), at 500 kb resolution. We transformed this dataset into 1 Mb resolution in our analysis. The epigenetic data of GM12878 were downloaded from ENCODE datasets: ENCFF167NBF (H3K27me3), ENCFF171MDW (H3K36me3), ENCFF803DJF (H3K79me2), ENCFF77 6OVW (H3K9me3) and ENCFF-180LKW (H3K27ac).

HiRES

After the quality control filtering in HiRES, a total of 399 cells from mouse brain and 7,469 cells from mouse embryos were obtained. All mouse brain cells remained were used for analysis, while for mouse embryos we focused on the two primary lineages comprising of 3,247 cells. The cell types were provided in the dataset, obtained from annotations on scRNA-seq. For the AML dataset, a total of 427 cells were obtained from 6 patients (named as PT01-PT06).

The scHi-C matrices were binned at 100 kb in mouse brains and embryos; at 500 kb in AML, unless otherwise stated. Only autochromosomes (chr1-19 for mouse; chr1-22 for human) were used for analysis. We ran scDIAGRAM on each chromosome separately. The preprocessing of pseudo-bulk Hi-C matrices in scDIAGRAM precisely followed the standardized pipeline implemented in cooltools Open2C et al. (2024).

We constructed metacells on two lineages and computed pseudotime on the early neuronal lineage (consisting of 2,296 cells). All cells in mitosis (M stage) were excluded when constructing metacells. The marker genes in mouse brains, embryos and AML were identified using Seurat Hao et al. (2021) with default parameters. Details can be referred to Supplementary Methods 1.9.

GAGE-seq

The quality control filtering of the GAGE-seq followed the same thresholds in Zhou et al. (2024). We only used the mouse brain cortex dataset in GAGE-seq. Cells were retained if it had: 1) at least 1,000 mouse RNA reads, 2) at most 1% of RNA coming from mouse mitochondria, 3) at least 50,000 mouse contact pairs, 4) at least 20 contact pair per 1 Mb on average on each mouse chromosome. The scHi-C matrices were also binned at 100 kb and autochromosomes (chr1-19) were used for analysis.

GAGE-seq may generate doublets. We used the DoubletDetect tool Gayoso et al. (2022) to detect and remove doublets. The BoostClassifier was trained with parameters n_iters=100, n_components=28. Doublets were then inferred by the trained classifier with thresholds p_thr=1e-2, v_thr=.3. After this, cells with more than 45K nonzero elements in the contact map were removed.

The cell types of GAGE-seq were obtained from clustering and annotation of scRNA-seq from GAGE-seq. The marker genes were also identified using Seurat Hao et al. (2021) with default parameters. Details can be referred to Supplementary Methods 1.9.

scRNA-seq

When computing the coefficient of variation (CV) to measure the transcriptional variability.

In scRNA-seq data, there is an inherent connection between the mean and standard deviation of read counts Hafemeister and Satija (2019). Thus we used the coefficient of variation (CV, the ratio of the standard deviation to the mean), to measure the transcriptional variability.

Due to the dropout events in the scRNA-seq, we utilized MAGIC Van Dijk and et al. (2018) to first impute the scRNA-seq data. Later when computing the correlation of compartments and RNA we also implemented the same imputation (Fig 3D, Supplementary Figure S18). In other cases, the original scRNA-seq was used.

1.9 Experimental details for the HiRES on AML

Patients source

We collected acute myeloid leukemia (AML) patient samples and their pathological results from the Department of Hematology at Peking University People's Hospital. All samples were obtained via bone marrow aspiration. The study was approved by the Ethics Committee of Peking University People's Hospital (2024PHB391-001). All patients signed informed consent forms as required.

Isolation of single cells

We isolated mononuclear cells from each patient's whole blood sample by using the Ficoll product (TBD, LTS1077). After density gradient centrifugation performed at 2000 × g for 20 minutes at 9:0 (brake off), cells were resuspended with 10 mL PBS (Gibco, 2124859). Then, we use 5 mL red blood cell lysis solution (Solarbio, 2312012) to remove red blood cells for 6 to 8 minutes on ice. The cells were resuspended with PBS and stained with flow cytometry antibodies CD34, CD117, CD45, 7-AAD, CD38 (Biolegend, 562577, 313218, 560777, 420403, 356605). After 20 minutes, cells were sorted by BD FACS Aria SORP to get CD34+CD117+CD38- hematopoietic stem and progenitor cells (HSPCs) for downstream HiRES experiments.

Library preparation and sequencing

Library was prepared strictly following HiRES protocolLiu et al. (2023). Sorted single cells were fixed with final 1.75% paraformaldehyde (PFA, ThermoFisher 28906) for 10 minutes at room temperature, followed by quenching with 2% BSA at a 10:1 volume ratio. Then, cells were resuspended in 200 μL ice-cold Wash Buffer (10 mM Tris pH 8.0, 10 mM NaCl, 0.1 mg/mL BSA (NEB B9000S)) supplemented with 20 μL protease inhibitor (Sigma P8340) and 2.6 μL Recombinant RNase Inhibitor. Each barcoded single-cell libraries were pooled and purified with 0.6× and 0.15× AMPure XP beads. The final libraries were sequenced with paired-end 150-bp reads on a NovaSeq X Plus (Illumina) platform.

1.10 More details in Data processing

Cell type annotating in GAGE-seq

In the GAGE-seq dataset from mouse brains, we used its scRNA-seq for cell type annotating. This dataset were generated from cells in the mouse cortex (8–9 weeks old), consisting of 3,143 cells after filtering.

We used the Seurat Hao et al. (2021) package. First we selected genes expressed in at least 10 cells. The expression data were then normalized using the 'NormalizeData' and 'ScaleData' functions with default parameters. Highly variable genes were identified with the 'FindVariableFeatures' function. PCA analysis was performed with the 'RunPCA' function with npcs=50 PCs. The Louvain clustering was performed with functions 'FindNeighbors' and 'FindClusters' using 20 neighbors, the first 27 PCs, the euclidean distance as the metric, and a resolution of 3. Then we run UMAP with 'RunUMAP' on the first 25 PCs to generate cell embedding and clustering.

After clustering we generated 29 clusters. Using the marker genes from the original paper Zhou et al. (2024) (genes *Slc17a7*, *Gad1*, *Slc7a10*, *Cspg4*, *Mag*, *Apod*, *Cx3cr1*), we divided these 29 clusters into three major lineages in the mouse cortex: 16 excitatory neuron subtypes, 8 inhibitory neuron subtypes and 5 glial cell subtypes. Each lineage exhibited unique marker gene expressions. In later studies we would compare the transcription (RNA) and genome organization (Hi-C) between the excitotory and inhibitory lineage.

Detecting markers from scRNA-seq

In the HiRES data from mouse brains, we have cell type annotation provided by the authors, with 7 cell types in total (excitatory neuron cluster 1-3, Ex1-3; inhibitory neuron cluster 1-2, In1-2; astrocyte, Ast; oligodendrocyte, Oli). Since the compartmental enrichments were not that obvious and quite noisy between cell subtypes (both for scDIAGRAM and scA/B), we here focused on 4 major cell types: Ex, In, Ast and Oli. We just utilized these cell types and used the Seurat "FindAllMarkers" function with "only.pos=T" parameter. Thus for each cell type, we used the remaining cell types as control and only detected those up-regulated markers in this cell type. All the other parameters were the default parameters. In Supplementary Figure S17, we selected the top 500 markers for each cell type and computed

their averaged scCompartments for each method.

In the GAGE-seq from mouse brains, after cell embedding and cell type annotating of the scRNA-seq, we only focused on the two large groups of cells, consisting of excitatory and inhibitory neurons. So we just used the Seurat "FindMarker" function to detect up-regulated markers in inhibitory neurons. Here we used the parameters "p_val_adj < 0.05" and "min.pct $= 0.01$". In this dataset we used the "MAST" test instead of the default "wilcox" test. All the other parameters were the default parameters.

In the HiRES data from the developing mouse embryos, we detected stage-specific markers for each stage, overlooking their cell types. We still used the the Seurat "FindAllMarkers" function with "only.pos=T" parameter, but we used the stages to group cells. So for each stage, we used the remaining stages as control and detected up-regulated markers for this stage. All the other parameters were the default parameters.

Embedding using scHi-C data

These embeddings were only used in Supplementary Figure S11A for UMAP visualization.

In the HiRES data from adult mouse brains, we compared different methods of cell embeddings based on scHi-C data (Supplementary Figure S11A).

First we used scCompartments from scDIAGRAM and scCpG for cell embedding. For scDIAGRAM we used the real-valued Ncut. First we computed PCA and selected the first 15 PCs. Then we run UMAP on these PCs, with the parameter "n_neighbors=10" and "min_dist=0". All the other parameters were set as default.

For scCpG we first performed a rank normalization into [0,1] after concatenating results from chr1-19. Then we did the same things as above. Took the first 15 PCs from PCA and fed them into UMAP with exactly the same parameters.

Then we used scHiCluster imputed matrix for cell embedding. For scHiCluster, we just utilized the flattened imputed matrices for each chromosome, and only considered contacts between pairs of loci located within 10Mb on the genome. We took the first 50 PCs for each intrachromsomal Hi-C matrix, then we concatenated them and took the first 20 PCs again and fed them into UMAP with the same parameters.

Higashi automatically generated embeddings. We just used its embeddings and fed into UMAP with the same parameters.

Metacell construction

In the HiRES data from developing mouse embryos, to decrease noise in single-cell data, we generated metacells for downstream analysis, following the same steps in Liu et al. (2023).

We defined single cells with similar RNA profiles as a metacell. Specifically, we first selected non-M phase single cells from the embryo dataset, generating 3,217 cells in total. We normalized these cells using "SCTransform" by Seurat, performed PCA dimensionality reduction, and used the first 25 principal components at a resolution of 35 to cluster single cells into metacells. Metacells with fewer than 5 cells were discarded in subsequent analyses. We obtained a total of 167 metacells for the embryonic data, with each metacell consisting a median of 18 single cells. The metacell's RNA profiles or compartments were generated by the mean of RNA expressions and scCompartments, for all single cells in this metacell.

For clustering of metacells, we used Seurat function "ScaleData" to normalize the metacell RNA profiles and performed PCA by "RunPCA" function with default parameters on top 2,000 variable features identified by "FindVariableFeatures" function. The cell typing of a metacell was carried out through a majority vote by all single cells within the metacell.

Pseudotime inference

For the HiRES data from developing mouse embryos, both the UMAP embedding of original scRNA-seq and metacell RNA-seq exhibited two differentiation trajectories, the neural trajectory (EN) and the mesenchymal trajectory (MLM), stemming from epiblast and primitive streak (EPI).

We performed pseudotime inference using Monocle3 Cao et al. (2019), based on the above UMAP embedding of metacells. We focused on the EN lineage and chose the EPI as starting points. Then we applied monocle3 to compute the pseudotime with default parameters.

2 Supplementary Results

2.1 Simulation via downsampling high coverage scHi-C data

We then compared scDIAGRAM with 3D structure modeling from Hickit by downsampling the Dip-C dataset Tan et al. (2018), a high-coverage scHi-C dataset, using 14 GM12878 cells on chr1 at 500 kb resolution. The original data was downsampled from 50% to 5%. On the original data, the compartments generated by scDIAGRAM were consistent with the 3D structure modeling from Hickit (median intersection 0.85, Spearman correlation 0.7). scHiCluster produced a median intersection of 0.7 and a Spearman correlation of 0.45, with larger variance.

Upon downsampling, scDIAGRAM outperformed both scHiCluster and Higashi in terms of intersection and Spearman correlation (Supplementary Figure S23). At low sampling rates (below 10%), scDIAGRAM also outperformed Hickit (Supplementary Figure S23). Furthermore, across different sample rates, the intersection and correlation for scDIAGRAM barely decreased, demonstrating the robustness of our method to variations in sequencing depth.

In contrast, scHiCluster exhibited limited imputation benefits, with global Pearson and Spearman correlations always below 0.6. This suggests that 3D modeling and scHiCluster may generate quite different imputed matrices from the same scHi-C data (Supplementary Figure S23B), which limits their utility in certain applications. Higashi produced better imputation results than scHiCluster on this simulated dataset, as measured by global Spearman correlation; however, its performance still fell short of ideal (Supplementary Figure S23B).

2.2 Running time and memory usage

Supplementary Fig. S10 shows the runtime and memory usage of different methods as a function of the number of cells. All CPU-based experiments were performed on an Intel Xeon Platinum 8358 machine with 1024 GB of memory using 20 CPU cores. scGHOST and Higashi were additionally evaluated on the same CPU configuration as well as on a single NVIDIA A100 GPU.

Overall, scDIAGRAM exhibits competitive computational efficiency. Its runtime and memory usage are comparable to lightweight methods such as scA/B, while remaining substantially lower than those of more computationally intensive approaches, including Higashi and Hickit, especially as the number of cells increases.

2.3 Convergence of the MCMC procedure

We ran the MCMC sampler for 10,000 iterations for both bulk and single-cell Hi-C datasets, and repeated the procedure five times for each dataset. To assess convergence, we computed the Gelman–Rubin statistic (see Supplementary Methods for details), and the results are shown in Supplementary Fig. S24. Convergence diagnostics were evaluated using both bulk and single-cell Hi-C data at 1 Mb resolution with 20 change-points.

Because the Gelman–Rubin statistic is defined for univariate MCMC chains, we computed this statistic separately for each change-point location and then averaged the values across all change-points to obtain an overall convergence diagnostic. For bulk Hi-C data, the averaged statistic dropped below 1.05 after approximately 5,000 iterations, whereas for single-cell Hi-C data, convergence was reached after approximately 9,000 iterations, reflecting the higher noise level in single-cell data. In both cases, 10,000 iterations were sufficient to ensure satisfactory convergence.

2.4 Comparing Compsc-Ncut across datasets

To evaluate the comparability of the Compsc-Ncut metric, we examined compartmental strength across different datasets and cell types using this measure.

First, we compared adult mouse brain neurons profiled by HiRES and GAGE-seq at 1 Mb resolution with $K = 30$. Under these matched conditions, Compsc-Ncut values were comparable across the two laboratories, whereas conventional compartment strength metrics (CompScore) exhibited noticeable discrepancies (Supplementary Fig. S25A). This result highlights the improved cross-dataset stability of Compsc-Ncut.

We further examined Compsc-Ncut across different cell types within the same dataset. Using the HiRES dataset, we observed that Compsc-Ncut values were largely consistent across neuronal subtypes and developmental stages when analyzed separately. In contrast, comparisons across distinct biological

contexts revealed systematic differences: developing embryos exhibited stronger overall compartmentalization than adult neurons (Supplementary Fig. S25B), and AML cells showed stronger compartmental strength than normal GM12878 cells (Supplementary Fig. S25C).

2.5 Tradeoff of the CP number

In Supplementary Figure S26, we examined the trade-off between the CP number K, resolution, data noise, and the distribution of annotation block lengths. scDIAGRAM was tested on both single-cell and bulk Hi-C data from the same cell type, at resolutions of 100 kb, 500 kb, and 1 Mb. For each choice of K, we reported the final compartment annotations and computed their block lengths. We found that data with lower noise (e.g., bulk Hi-C) or coarser resolution (e.g., 500 kb and 1 Mb) required fewer CP numbers to reach stable annotations. Increasing K generally produced shorter compartment blocks; however, the overall distribution of compartment lengths was largely consistent across resolutions and noise levels, further supporting that compartments reflect genuine biological structures rather than algorithmic artifacts.

2.6 Robustness of thresholding value in scA/B

Our conclusions were robust with respect to the threshold used to binarize scA/B compartments. In Supplementary Figure S27, we computed compartments using thresholds of 0.4, 0.5, and 0.6. Both the heatmaps and pooled compartments remained consistent across thresholds. The Pearson correlation between the pooled compartments and bulk PCA was highest at a threshold of 0.5 and changed only slightly with other values.

References

Abdennur, N., & Mirny, L. A. (2020). Cooler: scalable storage for Hi-C data and other genomically labeled arrays. *Bioinformatics*, *36*(1), 311–316. Retrieved from https://doi.org/10.1093/bioinformatics/btz540 DOI: 10.1093/bioinformatics/btz540

Bintu, B., Mateo, L. J., Su, J.-H., & et al. (2018). Super-resolution chromatin tracing reveals domains and cooperative interactions in single cells. *Science*, *362*, eaau1783.

Cao, J., Spielmann, M., Qiu, X., & et al. (2019). The single-cell transcriptional landscape of mammalian organogenesis. *Nature*, *566*, 496-502.

Gayoso, A., Shor, J., Carr, A. J., & et al. (2022). Jonathanshor/doubletdetection: doubletdetection v4.2. *Zenodo*. Retrieved from https://doi.org/10.5281/zenodo.6349517

Hafemeister, C., & Satija, R. (2019). Normalization and variance stabilization of single-cell Rna-seq data using regularized negative binomial regression. *Genome Biology*, *20*, 1-15.

Hao, Y., Hao, S., Andersen-Nissen, E., & et al. (2021). Integrated analysis of multimodal single-cell data. *Cell*, *184*, 3573-3587.e29.

Kent W.J, F. T., Sugnet CW., & et al. (2002). The Human Genome Browser at UCSC. *Genome Res.*, *12*(6), 996-1006.

Kim, H.-J., Yardımcı, G. G., Bonora, G., & et al. (2020). Capturing cell type-specific chromatin compartment patterns by applying topic modeling to single-cell Hi-C data. *PLoS Comput. Biol.*, *16*, e1008173.

Liu, Z., Chen, Y., Xia, Q., & et al. (2023). Linking genome structures to functions by simultaneous single-cell Hi-C and RNA-seq. *Science*, *380*, 1070-1076.

Nagano, T., Lubling, Y., Várnai, C., & et al. (2017). Cell-cycle dynamics of chromosomal organization at single-cell resolution. *Nature*, *547*, 61-67.

Newman, M. E. J. (2013). Spectral methods for community detection and graph partitioning. *Phys. Rev. E*, *88*, 042822.

Open2C, Abdennur, N., Abraham, S., & et al. (2024, 05). Cooltools: Enabling high-resolution Hi-C analysis in python. *PLOS Computational Biology*, *20*(5), 1-16. Retrieved from https://doi.org/10.1371/journal.pcbi.1012067

Su, J.-H., Zheng, P., Kinrot, S. S., & et al. (2020). Genome-scale imaging of the 3D organization and transcriptional activity of chromatin. *Cell*, *182*, 1641-1659.

Tan, L., Xing, D., Chang, C.-H., Li, H., & Xie, X. S. (2018). Three-dimensional genome structures of single diploid human cells. *Science*, *361*, 924-928.

Van Dijk, D., & et al. (2018). Recovering gene interactions from single-cell data using data diffusion. *Cell*, *174*, 716-729.

Zhan, Y., Musella, F., & Alber, F. (2024). Prediction of single-cell chromatin compartments from single-cell chromosome structures by maxcomp. *bioRxiv*. Retrieved from https://www.biorxiv.org/content/early/2024/07/04/2024.07.02.600897 DOI: 10.1101/2024.07.02.600897

Zhang, R., Zhou, T., & Ma, J. (2022). Multiscale and integrative single-cell Hi-C analysis with Higashi. *Nat. Biotechnol.*, *40*, 254-261.

Zheng, Y., & Keleş, S. (2020). Freehi-c simulates high-fidelity hi-c data for benchmarking and data augmentation. *Nature Methods*, *17*, 37-40.

Zhou, T., Zhang, R., Jia, D., & et al. (2024). GAGE-seq concurrently profiles multiscale 3D genome organization and gene expression in single cells. *Nat. Genet.*, *56*, 1701-1711.

3 Supplementary Figures

Datasets	CP number used			
	Pseudo bulk (1 Mb)	Single-cell (1 Mb)	Pseudo bulk (100 kb)	Single-cell (100 kb)
GAGE (neuron, Zhou et al. (2024))	20	40	20	100
GM12878 (Kim et al. (2020))	20	40	30	80
HiRES (neuron, Liu et al. (2023))	30	60	60	100
HiRES (embryo, Liu et al. (2023))	30	80	60	120
HiRES (AML)	20	60	40	100

Supplementary Fig. S1. CP numbers used in this study.

We list the CP numbers used for each dataset at various resolutions, both at the pseudo-bulk and single-cell levels. Here, only the CP numbers for the full chromosome 1 (the longest chromosome) are shown. For other chromosomes, we recommend selecting candidate CP sets using min{L/3,m}, where L is the total number of bins in the chromosome and m is the CP number used for chromosome 1.

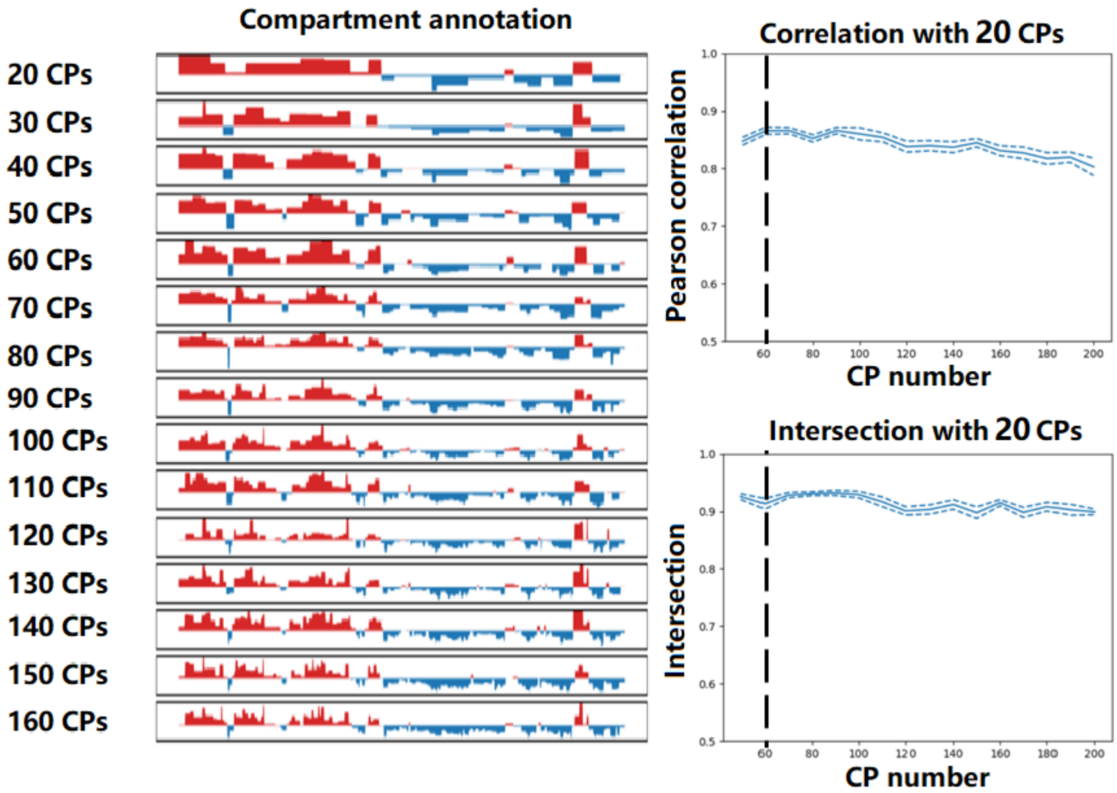

Supplementary Fig. S2: Robustness of scDIAGRAM to the choice of CP number on a real single cell (GAGE-seq, chr1, 100kb).

scDIAGRAM exhibited robustness to CP number selection. Testing on a single cell (GAGE-seq, chr1 100kb pre-centromere, not many CPs in bulk PCA), results stabilized at 20 CPs. Further increases minimally affected output, as seen in visual concordance and high correlation/intersection with the 20-CP results.

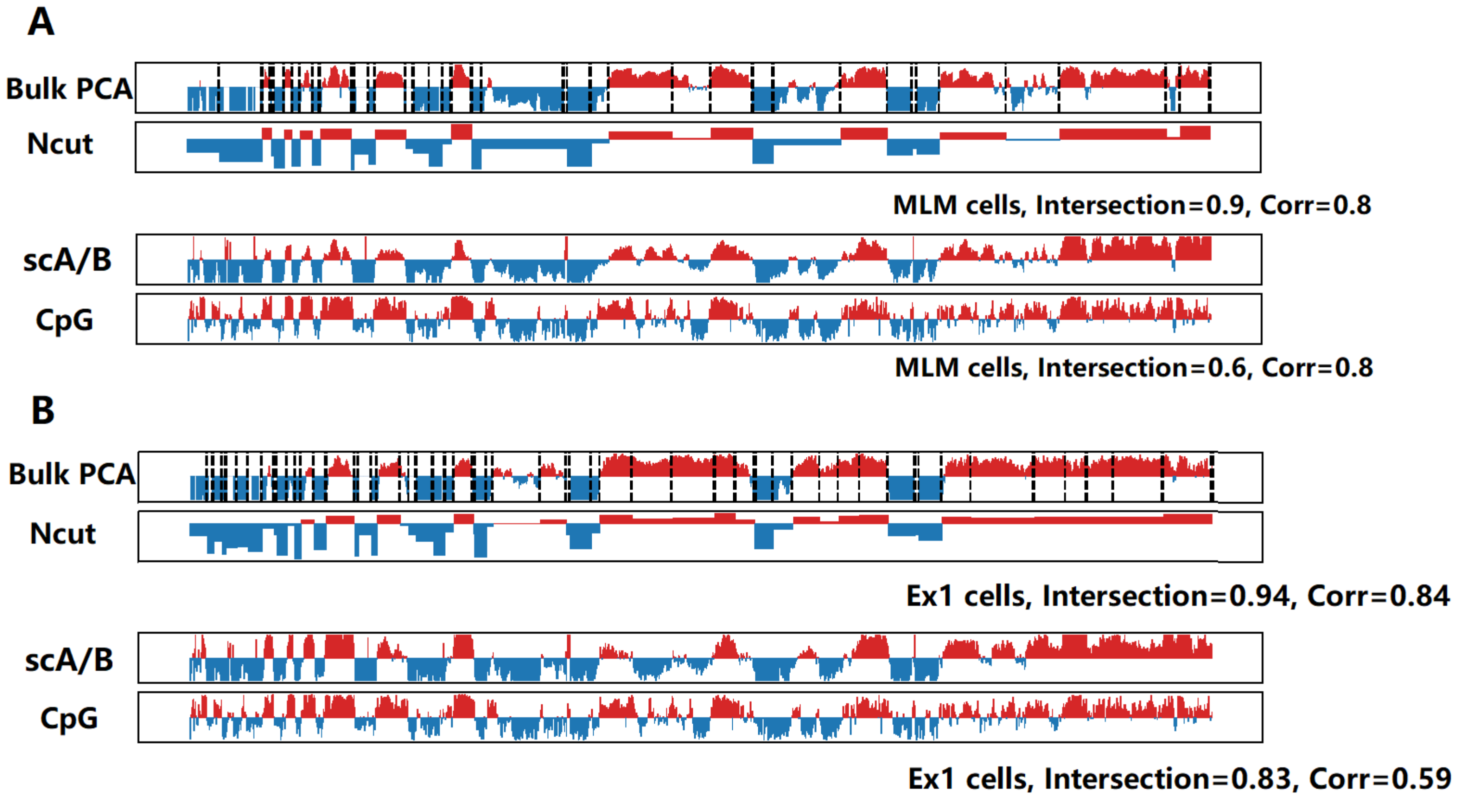

Supplementary Fig. S3. Compartment annotation of pseudo-bulk Hi-C (100kb, chr7) in mix late mesenchyme (MLM) and Ex1 cell types.

(A) From MLM pseudo-bulk Hi-C we computed bulk PCA (40 CPs, dashed lines), Ncut, scA/B, and CpG density (from the reference genome), with intersections/Pearson correlations between both Ncut and scA/B with bulk PCA.

(B) Same analyses as in (A), using the Ex1 pseudo-bulk Hi-C matrix.

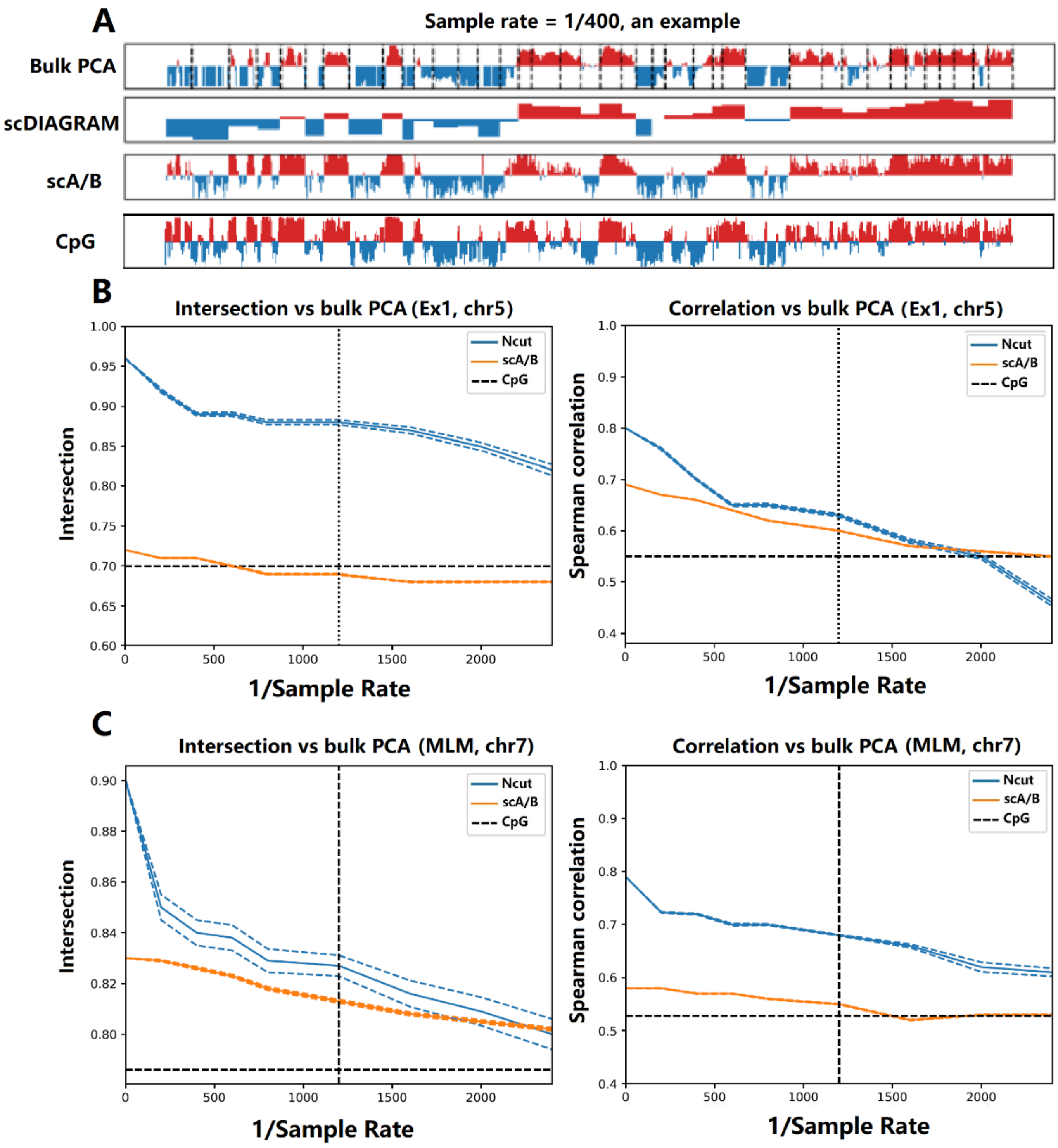

Supplementary Fig. S4. Performance evaluation of scDIAGRAM on downsampled pseudo-bulk Hi-C data.

(A) For Ex1 pseudo-bulk Hi-C at 100kb resolution (1/400 sampling rate), we compared compartments from scDIAGRAM and scA/B against the ground-truth bulk PCA (40 CPs), along with CpG density profiles.

(B-C) Intersection and Spearman's correlation for downsampled data from the (B) Ex1 (chr5) and (C) MLM (chr7) cell type, with vertical dashed lines indicating real scHi-C sampling rates and horizontal lines showing CpG vs bulk PCA intersection/correlation levels. At the real scHi-C sampling rate 1/1200, scDIAGRAM was always better than scA/B. At the rate 1/2400, when the downsampled Hi-C matrices was too sparse, scDIAGRAM might be worse than scA/B as scA/B would approximate the CpG level.

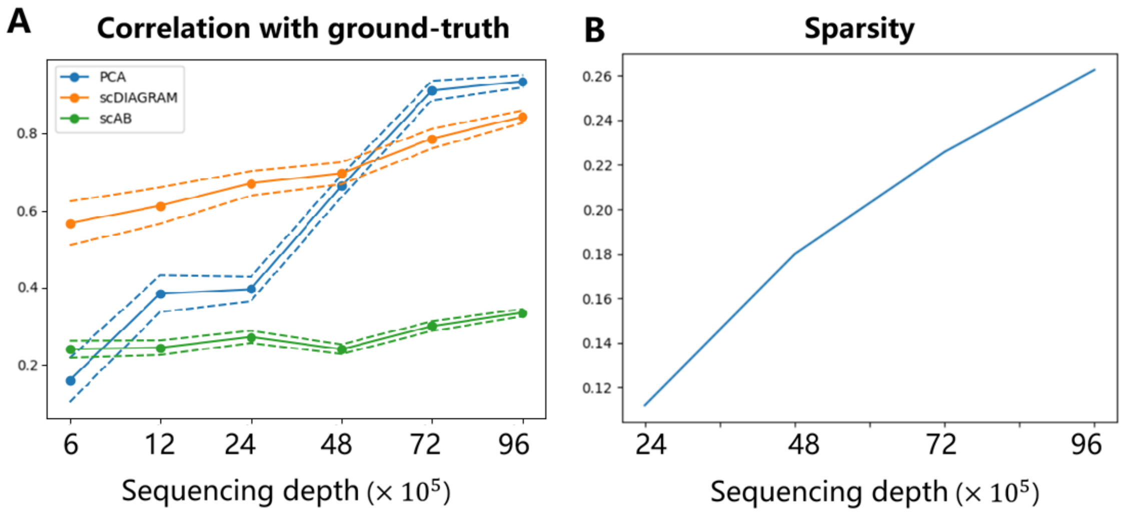

Supplementary Fig. S5. Comparison of PCA, scDIAGRAM, and scA/B on FreeHi-C simulated Hi-C data.

(A) FreeHi-C simulates in silico Hi-C matrices with varying sequencing depths from a bulk Hi-C matrix. The bulk PCA from the original data was used as the ground truth. Compartments generated by PCA, scDIAGRAM, and scA/B were compared on these simulated matrices across different sequencing depths. scDIAGRAM performed better than scA/B and PCA at low sequencing depths, highlighting its suitability for low-quality bulk Hi-C data or single-cell Hi-C data. This conclusion was consistent with those obtained from downsampling experiments. Error bar was computed for 10 repeats.

(B) Sparsity (fraction of non-zero elements) of the simulated Hi-C matrices at 10 kb resolution.

Datasets	Contact numbers per cell			
	Mean	Min	Median	Max
Nagano et al.(2017)	4.4 k	0.2 k	4.4 k	19.4 k
WTC-11 (Zhang et al. (2022))	14.5 k	2.2 k	14.7 k	25.3 k
Ramani et al. (2017)	0.9 k	0.0 k	0.7 k	9.5 k
4DN sci-Hi-C (Kim et al. (2020))	0.6 k	0.0 k	0.4 k	77.3 k
sn-m3c-seq (Lee et al. (2019))	14.0 k	0.4 k	15.5 k	37.9 k
HiRES (Liu et al. (2023))	24.4 k	4 k	23.6 k	42.8 k
GAGE-seq (Zhou et al. (2024))	26.6 k	0.1 k	23.4 k	202.8 k
Dip-C GM12878 (Tan et al. (2018))	43.9 k	32.2 k	43.4 k	67.5 k

Supplementary Figure S6. Statistics of the contact numbers per cell at 1Mb resolution of chr2.

Statistics of the contact numbers per cell at 1Mb resolution of chr2 for different datasets. The Nagano et al. data were mapped to the mm9 assembly. The Ramani et al. and sn-m3c-seq data were mapped to the hg19 assembly. HiRES and GAGE-seq were mapped to the mm10 assembly. Others were mapped to the hg38 assembly.

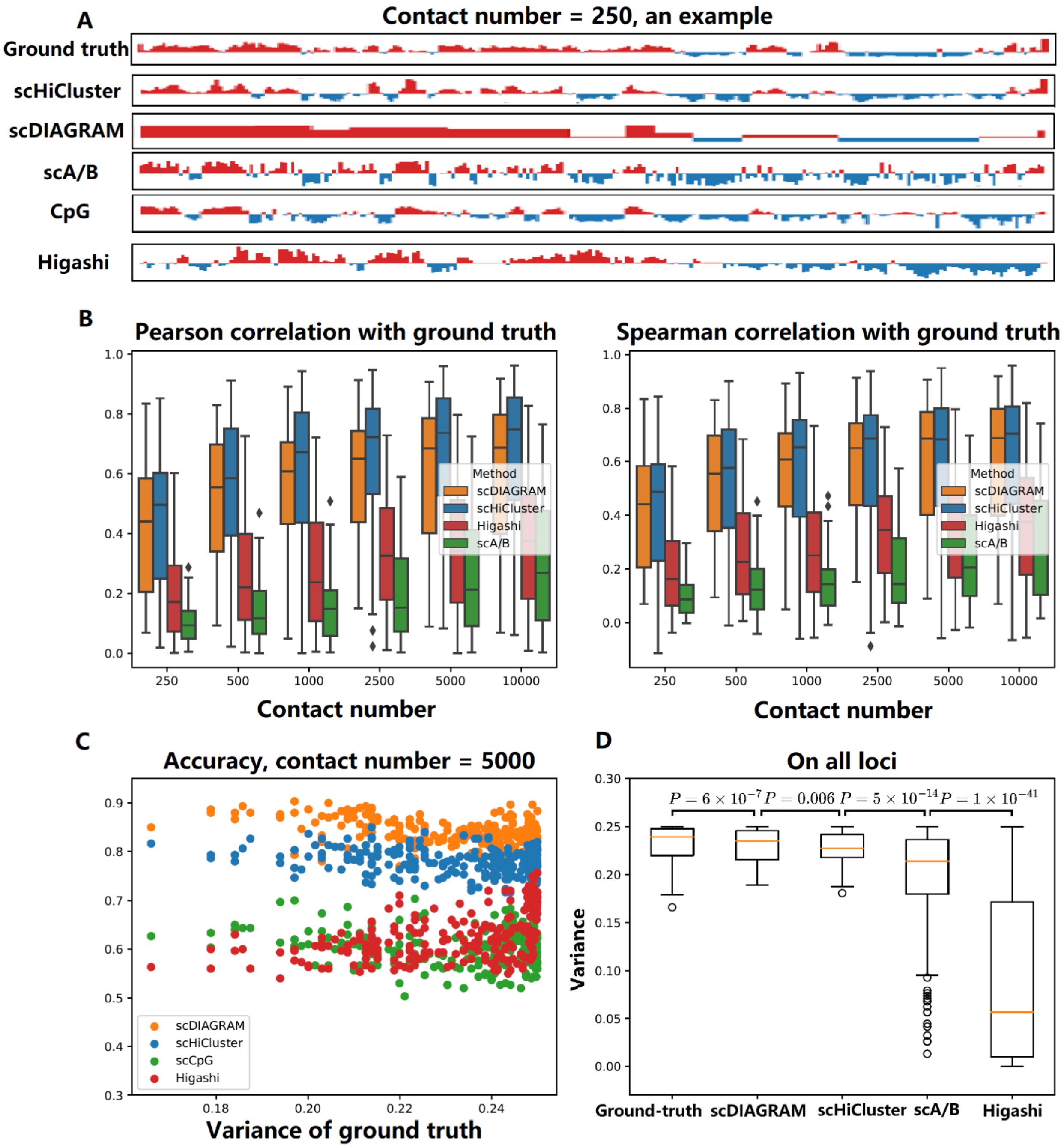

Supplementary Fig. S7. Performance on downsampled imaging data (Su et al. IMR-90, chr2).

(A) At 1Mb resolution (downsampled with 250 contacts), we showed the ground-truth bulk PCA compared with scHiCluster, scDIAGRAM, scA/B compartments and the CpG density.
(B) Correlation analysis revealed scDIAGRAM performed comparably to scHiCluster, while scA/B showed the lowest agreement with bulk PCA. Higashi performed slightly better than scA/B, but inferior to scDIAGRAM and scHiCluster.
(C) When downsampled at 5000 contacts, scDIAGRAM maintained the highest accuracy versus other methods. (D) scDIAGRAM exhibited greater heterogeneity than scHiCluster, Higashi and scA/B across all loci.

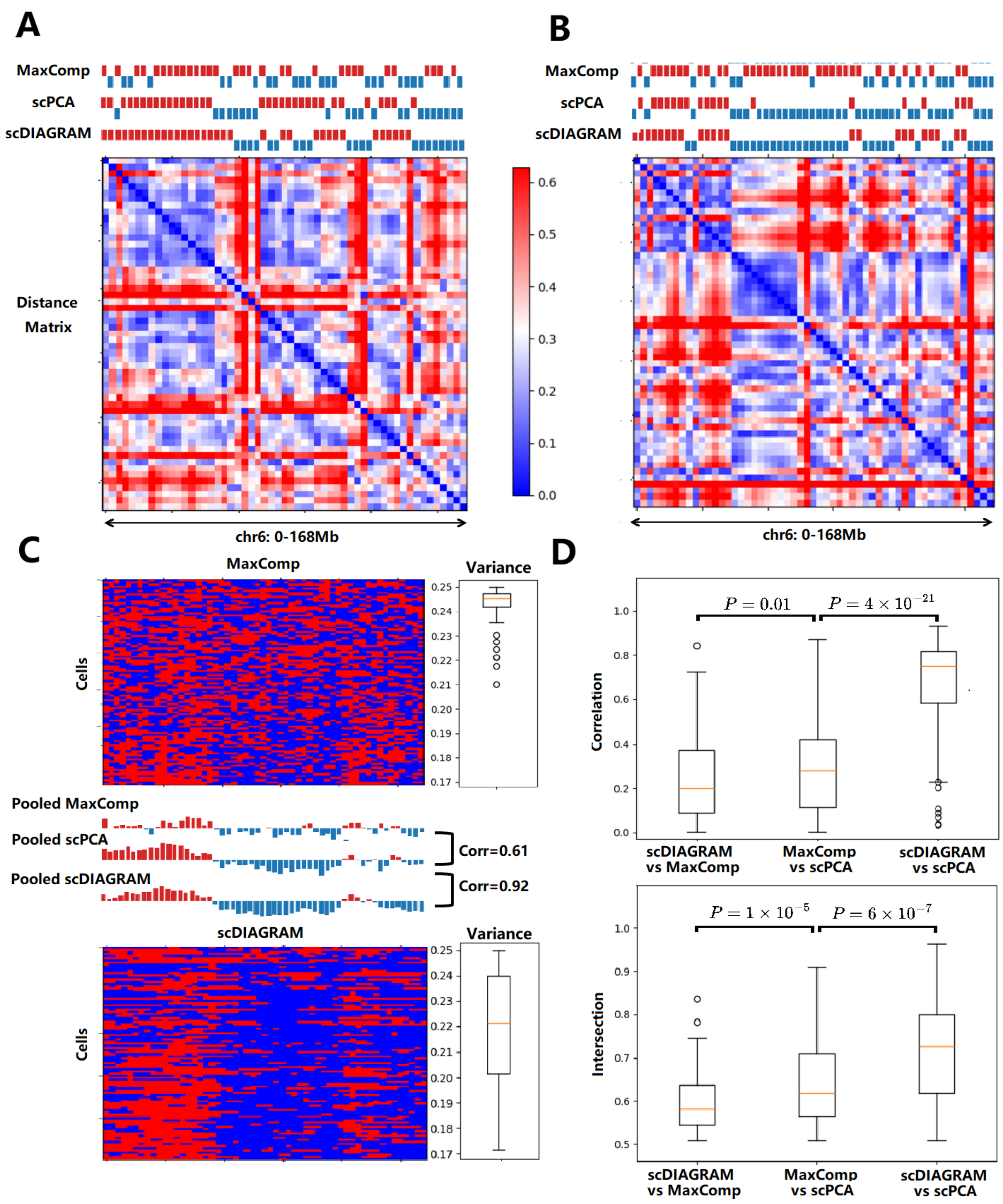

Supplementary Fig. S8. Comparison with MaxComp on the imaging data.

(A, B) Two examples of single-cell compartments obtained by MaxComp, scPCA, and scDIAGRAM. The corresponding distance matrices from the imaging data are also shown.

(C) Heatmaps of single-cell compartments annotated by MaxComp and scDIAGRAM for 100 cells, along with the pooled compartments from MaxComp, scPCA, and scDIAGRAM. Both the visual inspection and Pearson correlation indicate that the pooled scDIAGRAM compartments are closer to pooled scPCA than to MaxComp.

(D) Pearson correlation and binary intersection between each pair of methods for individual cells.

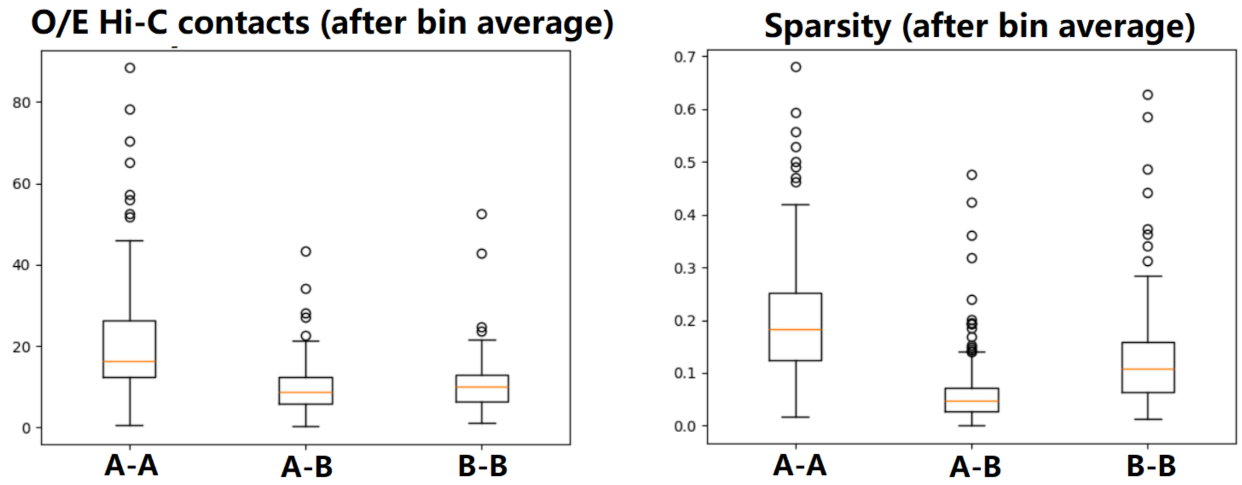

Supplementary Fig. S9. Compartment analysis on human GM12878 cells.
Median observed-over-expected (O/E) contact frequencies (A-A, A-B, B-B) from scDIAGRAM after bin averaging, with corresponding contact sparsities. The median was computed for nonzero values.

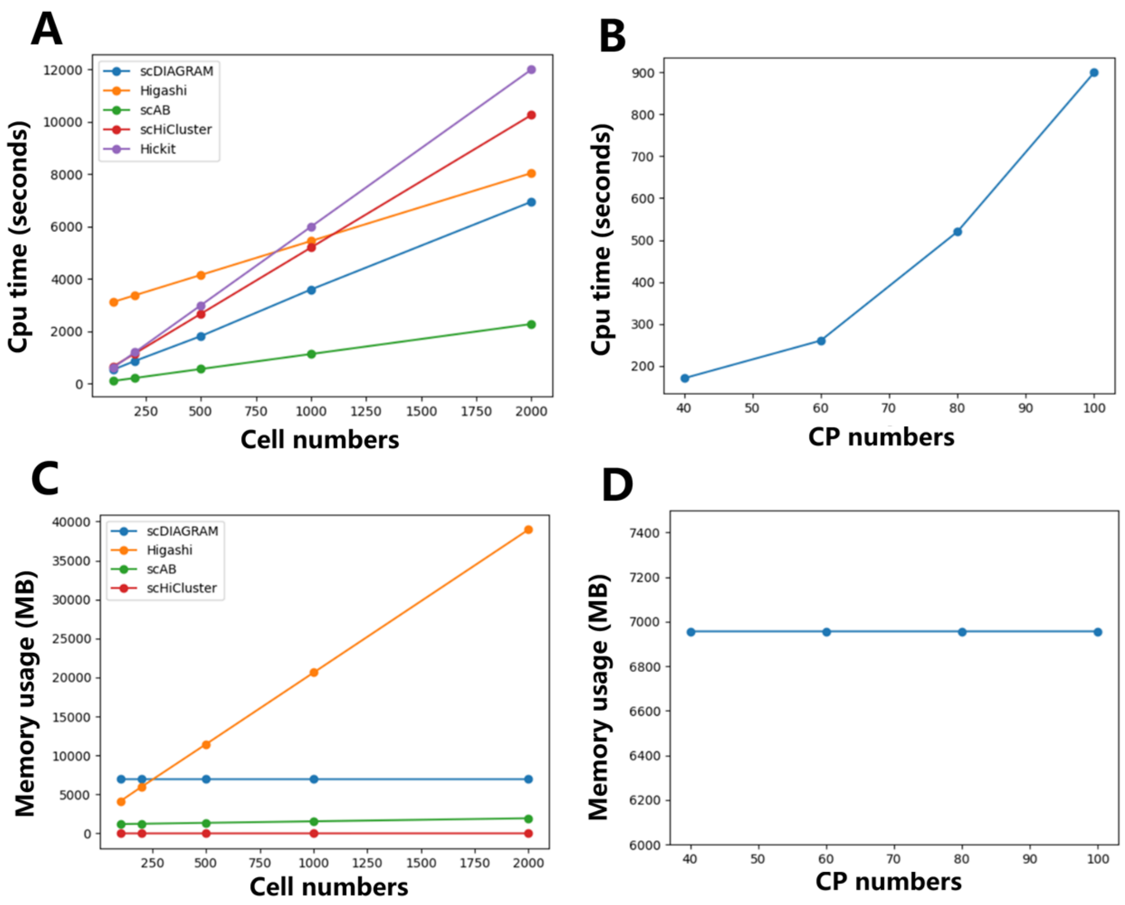

Supplementary Fig. S10. CPU time and memory usage for different methods across varying cell numbers and CP numbers.

(A) CPU time (seconds) for different methods with varying numbers of cells. All methods were run on Intel Xeon Platinum 8358 CPUs with 1024 GB RAM and 20 cores; Higashi additionally used one A100 GPU. scDIAGRAM was run with 80 CPs. scDIAGRAM demonstrated high scalability, second only to scA/B in CPU time.

(B) CPU time of scDIAGRAM at different CP numbers, tested on 200 cells to evaluate the effect of CP.

(C) Memory usage (MB) for each method. For Higashi, memory usage includes both CPU and GPU memory. Except for Higashi (which requires neural network training), all other methods process data one cell at a time, so their memory usage is independent of cell number.

(D) Memory usage of scDIAGRAM at different CP numbers.

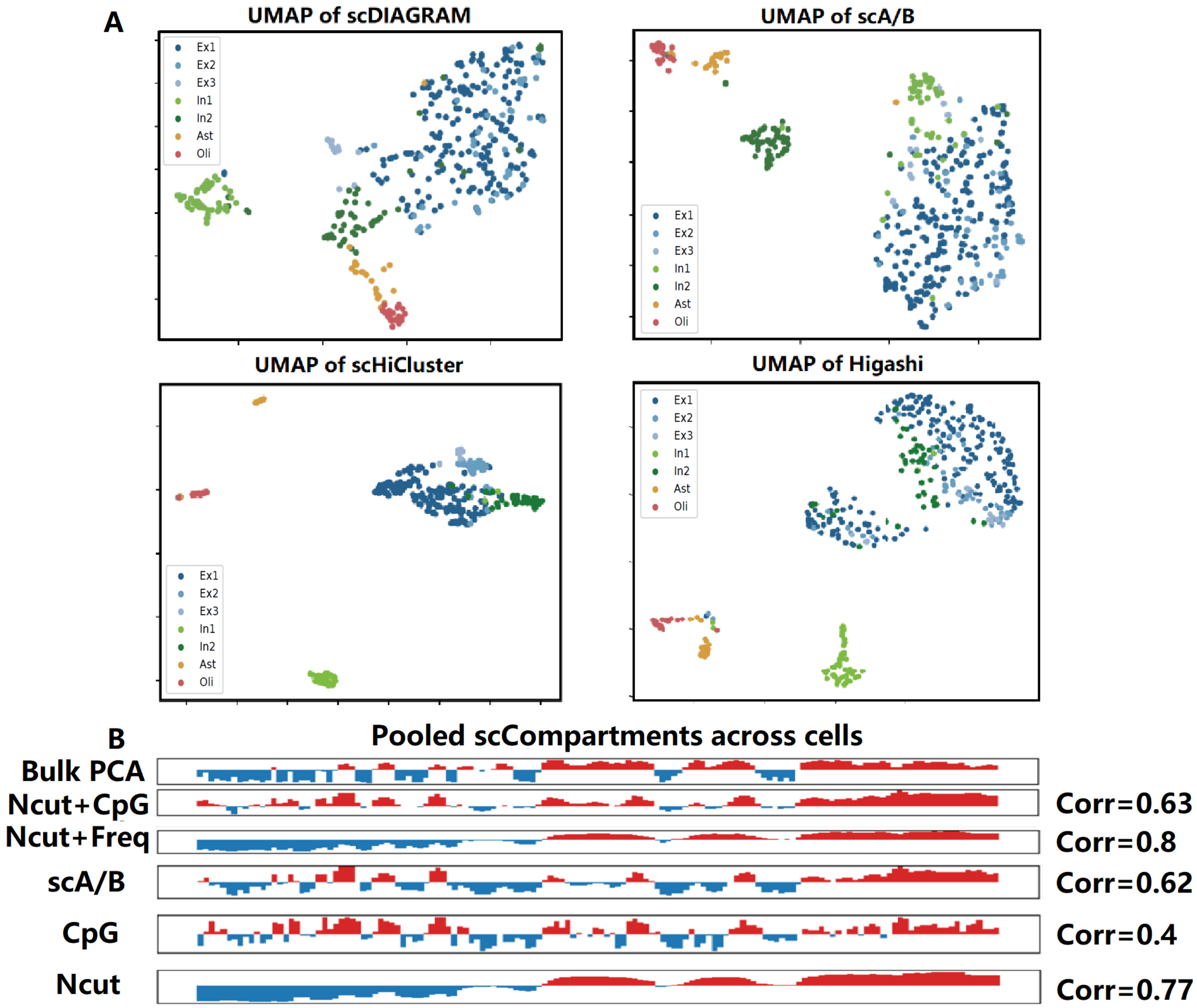

Supplementary Fig. S11. Embedding and Pooled compartments of the mouse brain neurons.

(A) UMAP embeddings of single cells using scDIAGRAM, scA/B, Higashi and scHiCluster, with scRNA-seq annotations (Ex1-3: excitatory neurons; In1-2: inhibitory neurons; Ast: astrocyte; Oli: oligodendrocyte). scDIAGRAM, scHiCluster and Higashi showed similar clustering patterns (In2 was more close to Ex1-3), while scA/B exhibited distinct patterns. (B) Pooled Ex1 scCompartments (chr7, 1Mb) compared to bulk PCA, with Pearson correlations calculated. Ncut, Ncut+Freq and Ncut+CpG compartments were studied alongside scA/B and CpG. Pooled scDIAGRAM (Ncut) correlated better with bulk PCA than scA/B. When combining Ncut with CpG, unlike in other analysis, it would reduce the correlation.

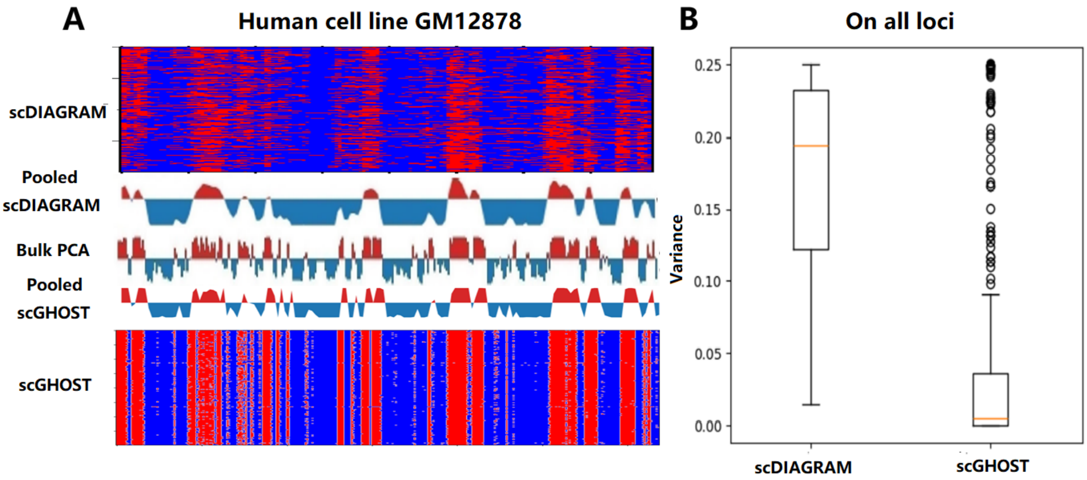

Supplementary Fig. S12. Comparison of scDIAGRAM and scGHOST using the human GM12878 cell line.

(A) Heatmaps of single-cell compartments for 300 GM12878 cells by scDIAGRAM and scGHOST. Pooled compartments from both methods are also shown, along with the bulk PCA for reference.

(B) Compartmental variance across cells for the two methods.

Both the heatmaps and variance plots indicate that scDIAGRAM exhibits greater heterogeneity than scGHOST.

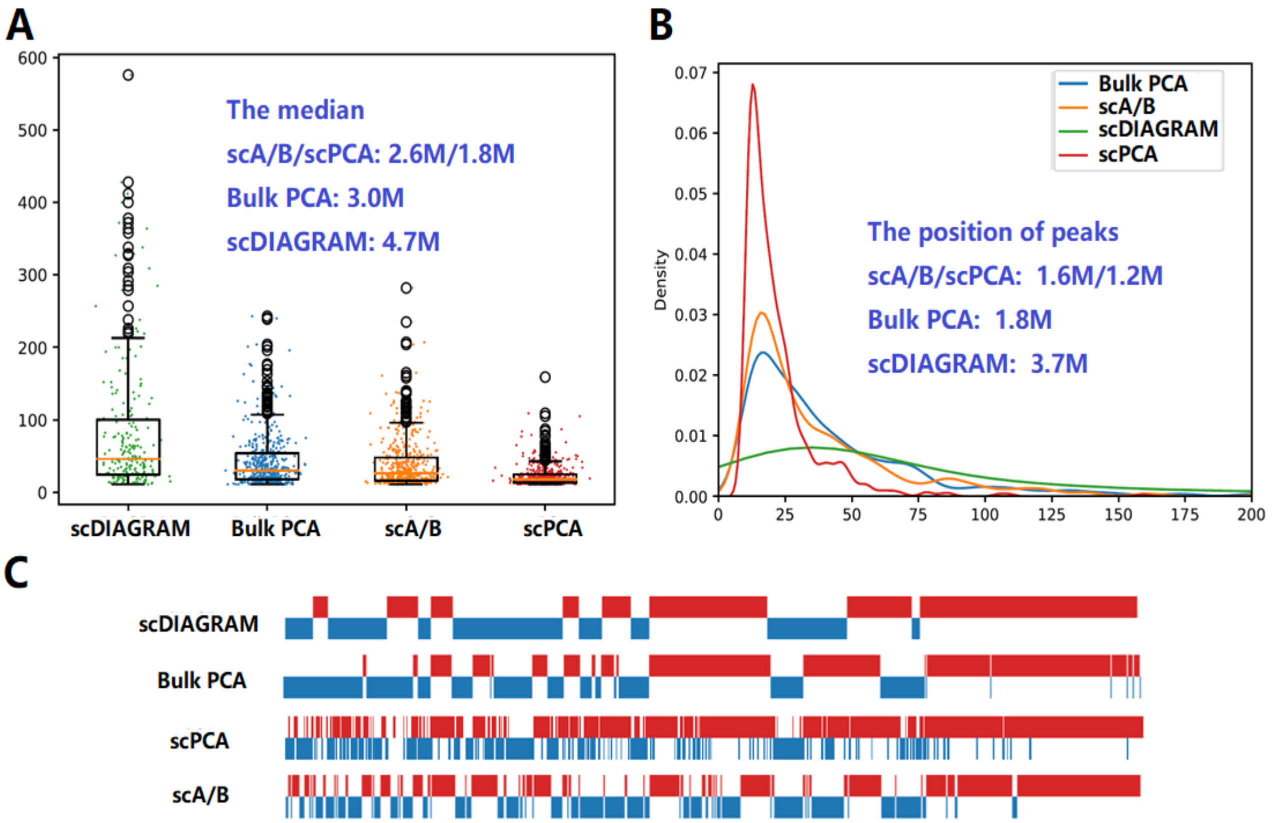

Supplementary Fig. S13. Compartmental length of the mouse brain neurons.
Results were generated from one cell on the whole genome at 1 Mb resolution.
(A) Boxplot comparing compartment lengths across methods, with scDIAGRAM showing closest median length to bulk PCA.
(B) Unimodal kernel density distributions revealed scDIAGRAM's peak position most closely matched bulk PCA.
(C) Example binary compartments demonstrating compartmental lengths for each method.

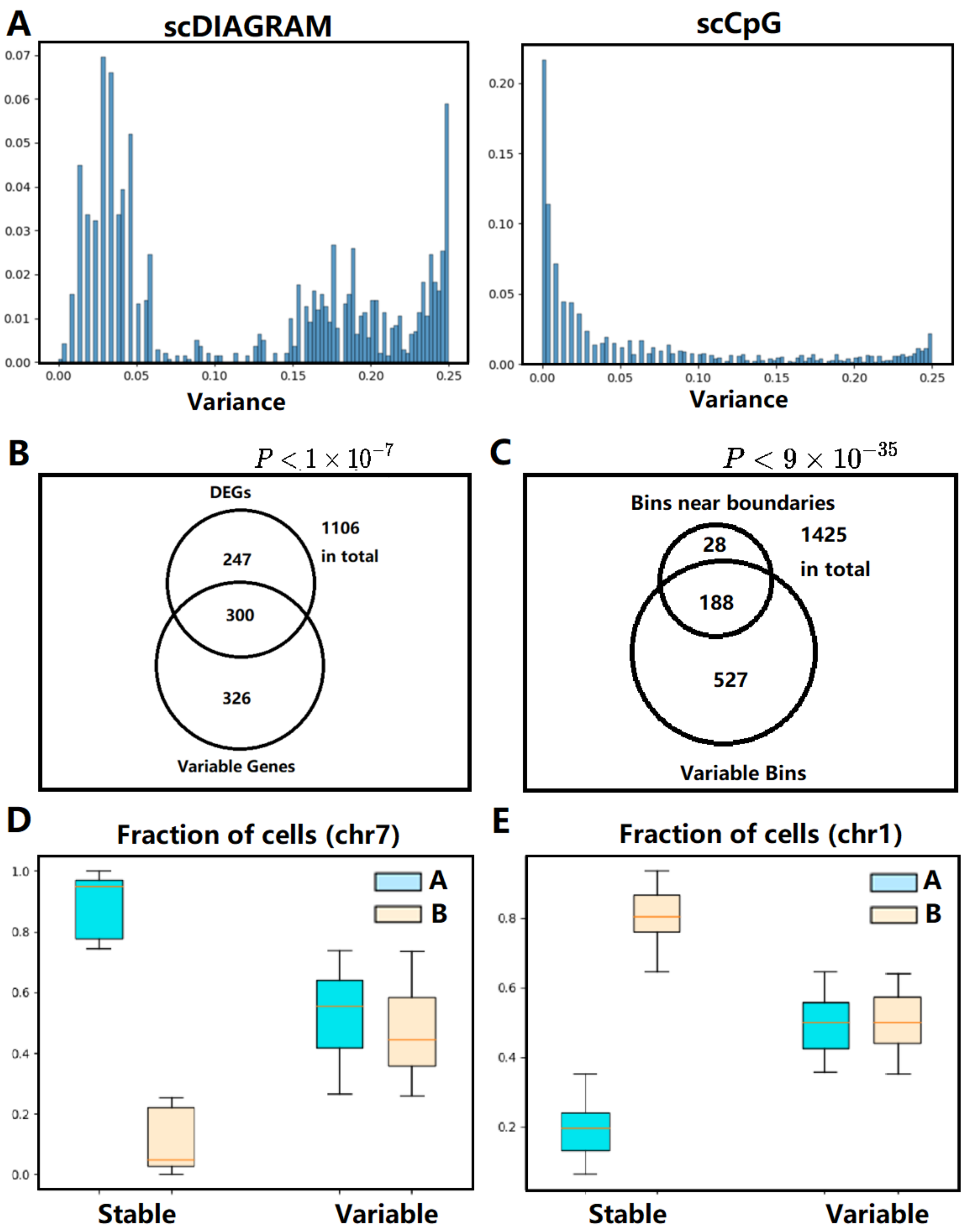

Supplementary Fig. S14. Stable/variable bins of the mouse brain neuron scHi-C data.

(A) Bimodal variance distributions (scDIAGRAM and scA/B) revealed stable and variable genomic loci.

(B) Compartment variability (for different cell types, across the dataset) correlated with cell-type-specific marker genes.

(C) Within Ex1 cells, the compartmental variability associated with compartmental

boundaries.

(D-E) Fraction of cells for stable/variable bins in A/B compartments, on different chromosomes. Variable regions distributed uniformly across A/B compartments, while stable regions predominantly occupy a single compartment. Here we used the top and lower 25-th percentile as the threshold to determine stable/variable regions.

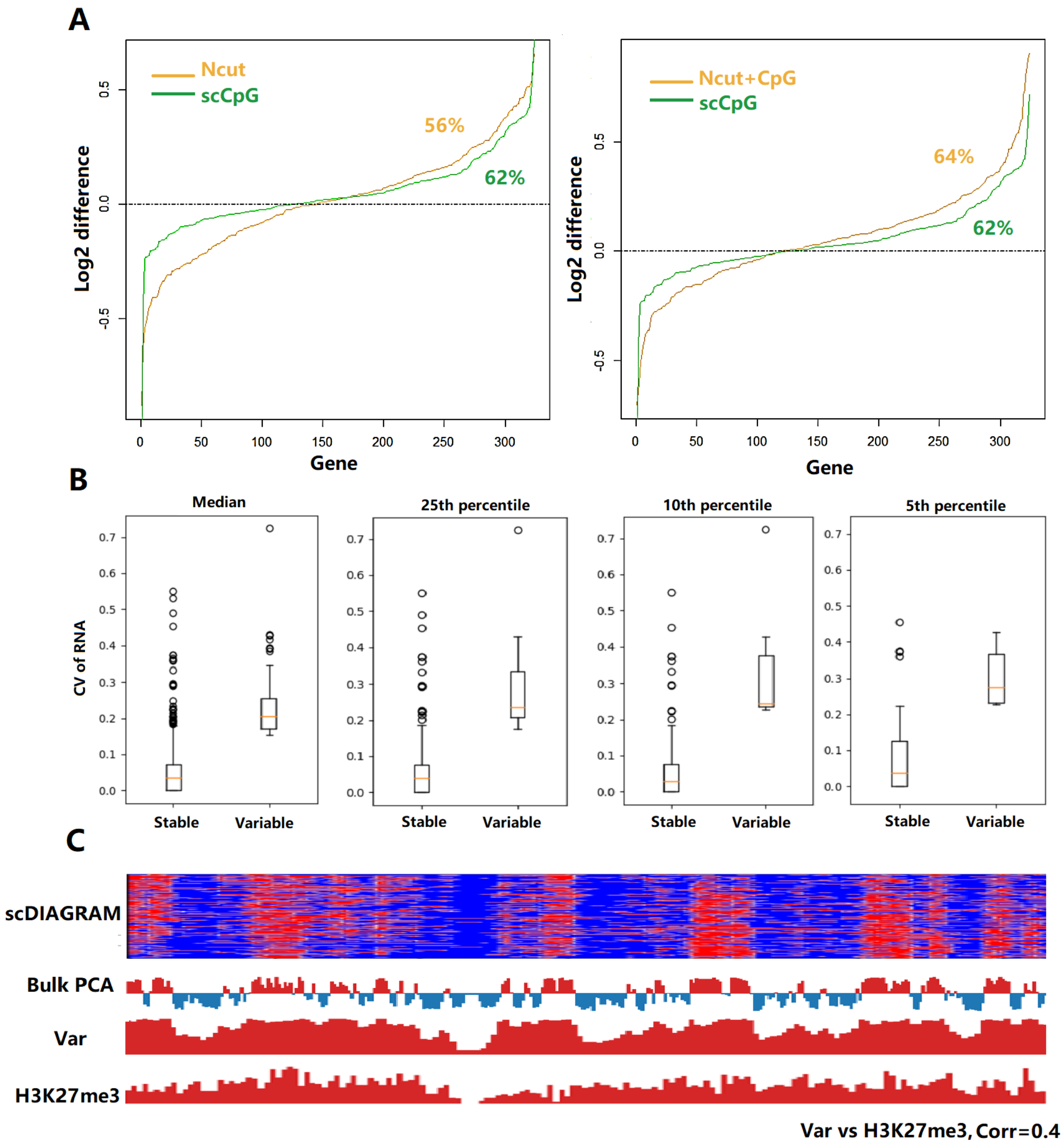

Supplementary Fig. S15. Linking single-cell compartments with RNA expressions in mouse brain neurons.

(A) In Ex1 cells, transcribing genes (UMI >10) showed more active compartments than silent genes (UMI <1). We took average of scCompartments across these states after normalized into [0,1], then we computed the log2 difference. We also computed the ratio of genes that was compartmental more activated in transcribing states. The ratio was comparable among these methods. Ncut typically generated larger compartmental difference compared with scA/B, indicating scDIAGRAM was more heterogenous.

(B) The robustness of our results when different thresholds were used. We computed the transcriptional variability (CV) for variable/stable regions, when different thresholds were used to determine the variable/stable groups.

(C) In GM12878 cells (chr7, 1Mb), we showed the heatmap from scDIAGRAM and bulk

PCA. Then we found scDIAGRAM's variance was correlated with H3K27me3, a mark enriched in B1 subcompartments and hence indicated the region's compartmental instability.

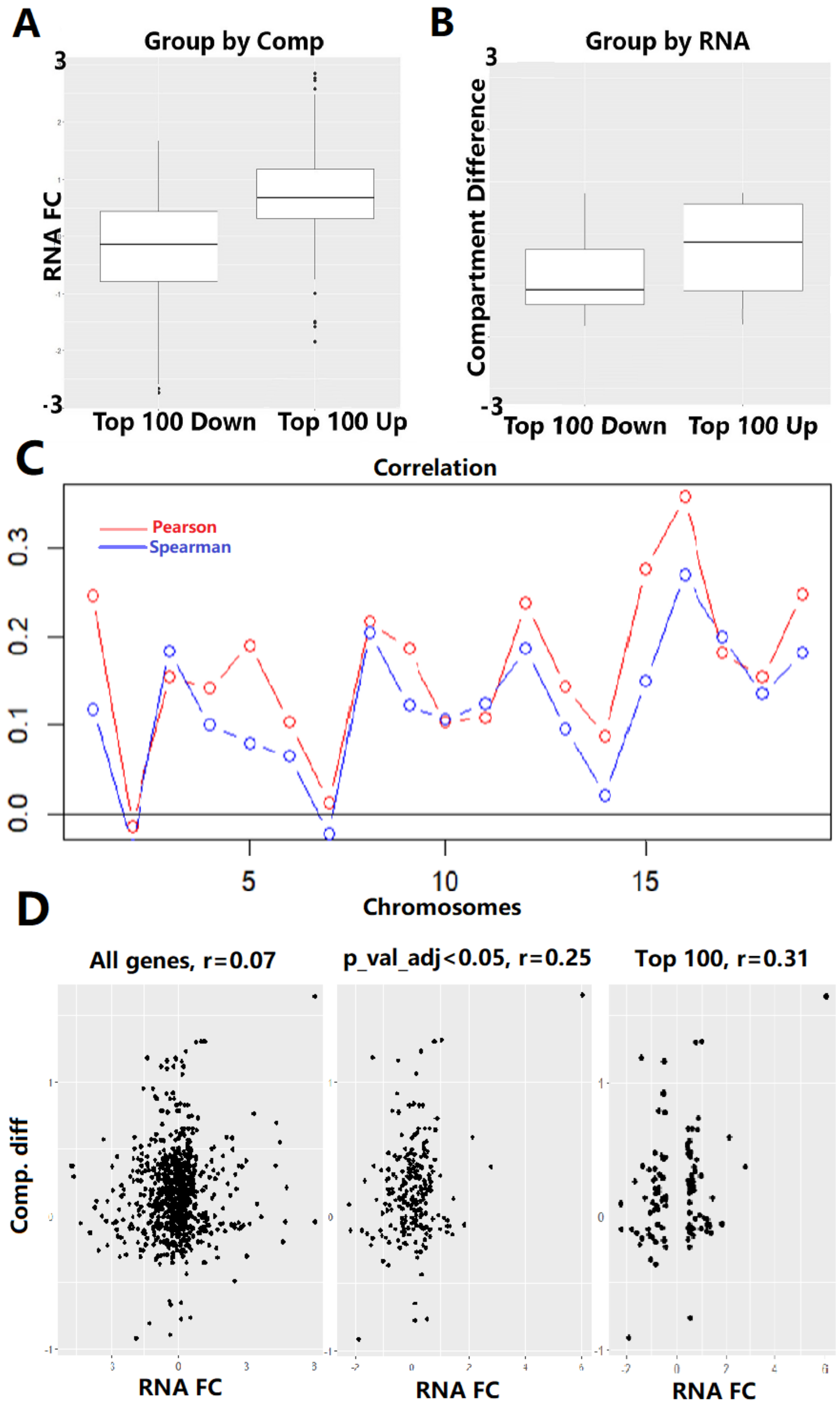

Supplementary Fig. S16. Compartment-RNA relationships between two cell types, using mouse brain neurons.

(A-B) Comparing the transcription and compartmental transition between Ex1 and Ast cells: (A) Top 100 compartment-activated genes (by scDIAGRAM) showed higher RNA

upregulation than top 100 inactivated genes, while (B) top 100 up-regulated genes exhibited compartmental activation than top 100 down-regulated genes, suggesting compartment changes may influence transcription. In (A) we observed larger difference between two groups, indicating compartmental changes as a potential cause for RNA transcription.

(C) Chromosome-wide analysis revealed positive RNA-compartment correlations (comparing RNA fold change with compartmental differences) between excitatory/inhibitory neurons, except for chr2/chr7.

(D) In chr1, stronger correlations emerged with increasingly stringent gene selection: all genes expressed in at least 1% cells (760 genes) → significant genes (230 genes, adjusted $p<0.05$) → top 100 genes ordered by fold-change, highlighting compartment-RNA coupling.

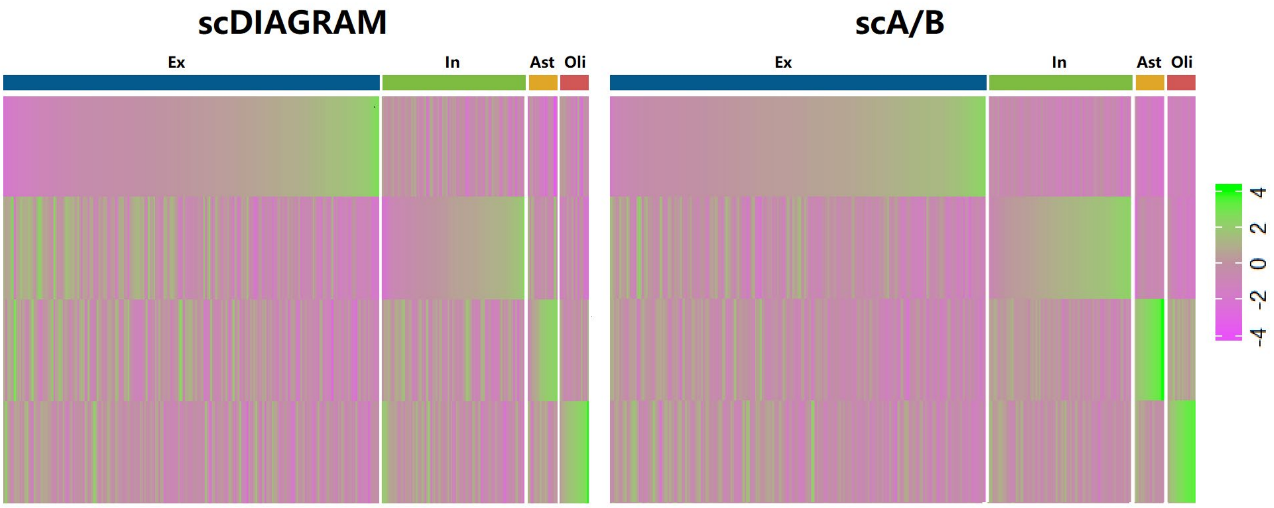

Supplementary Fig. S17. Compartment values on cell-type-specific marker genes (mouse brain neurons).

Mean compartmental values for top 500 marker genes across 4 cell types. Each column referred to a single cell (ordered by their cell types and the compartmental enrichment at the corresponding markers). Each row referred to a marker gene set. We used real-valued compartments from scDIAGRAM and each row was z-score normalized after averaging across markers.

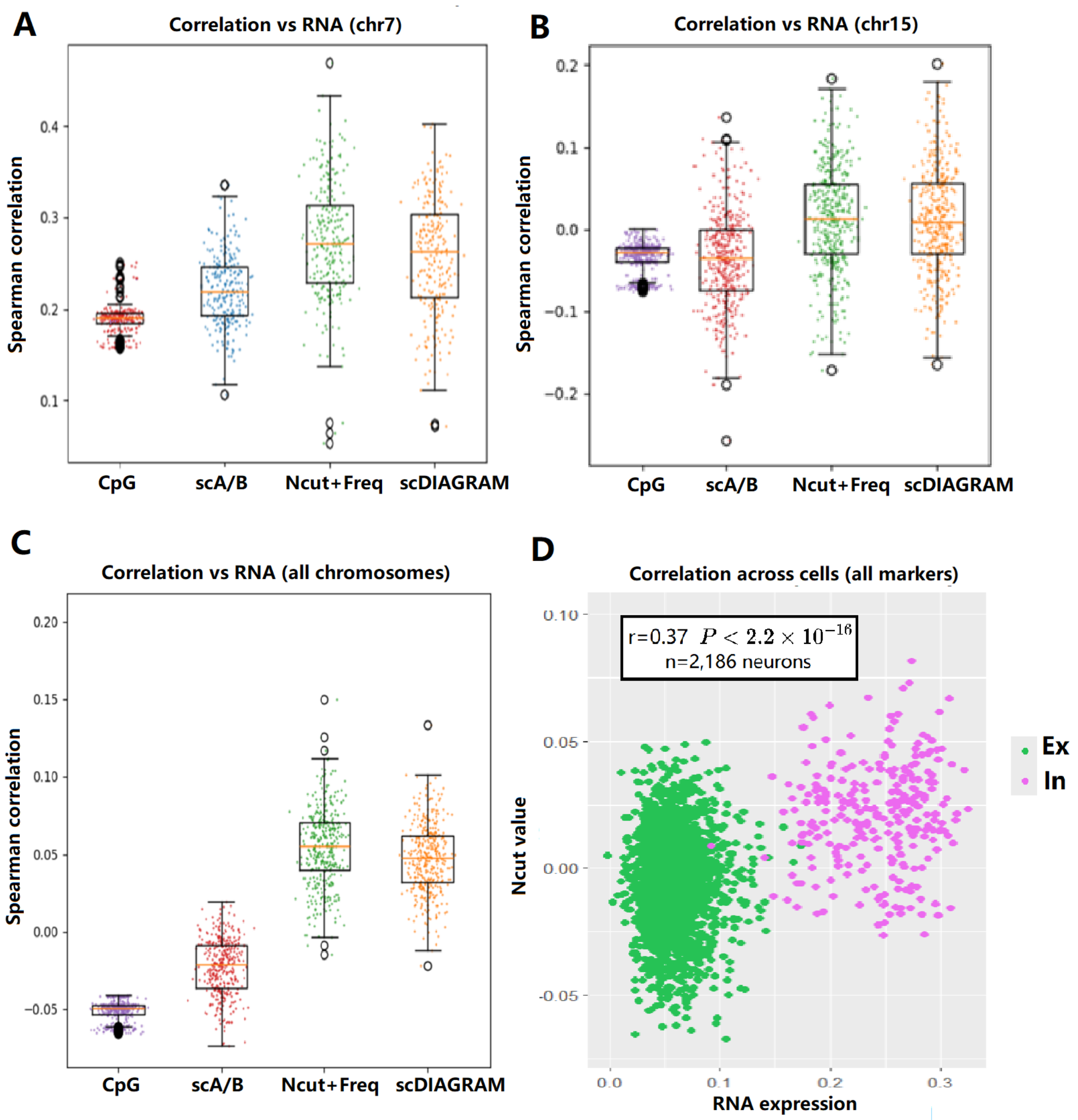

Supplementary Fig. S18. Compartment-RNA correlations in mouse brain neurons.
(A-C) used the HiRES dataset.
(A-B) Spearman correlations between RNA expressions and real-valued compartments from scDIAGRAM, on chr7 and chr15.
(C) Spearman correlations for all chromosomes.
(D) Significant positive correlation (r=0.37) between scDIAGRAM compartments and marker gene expression (n=2,186 Ex vs. In neurons, using the GAGE-seq dataset).

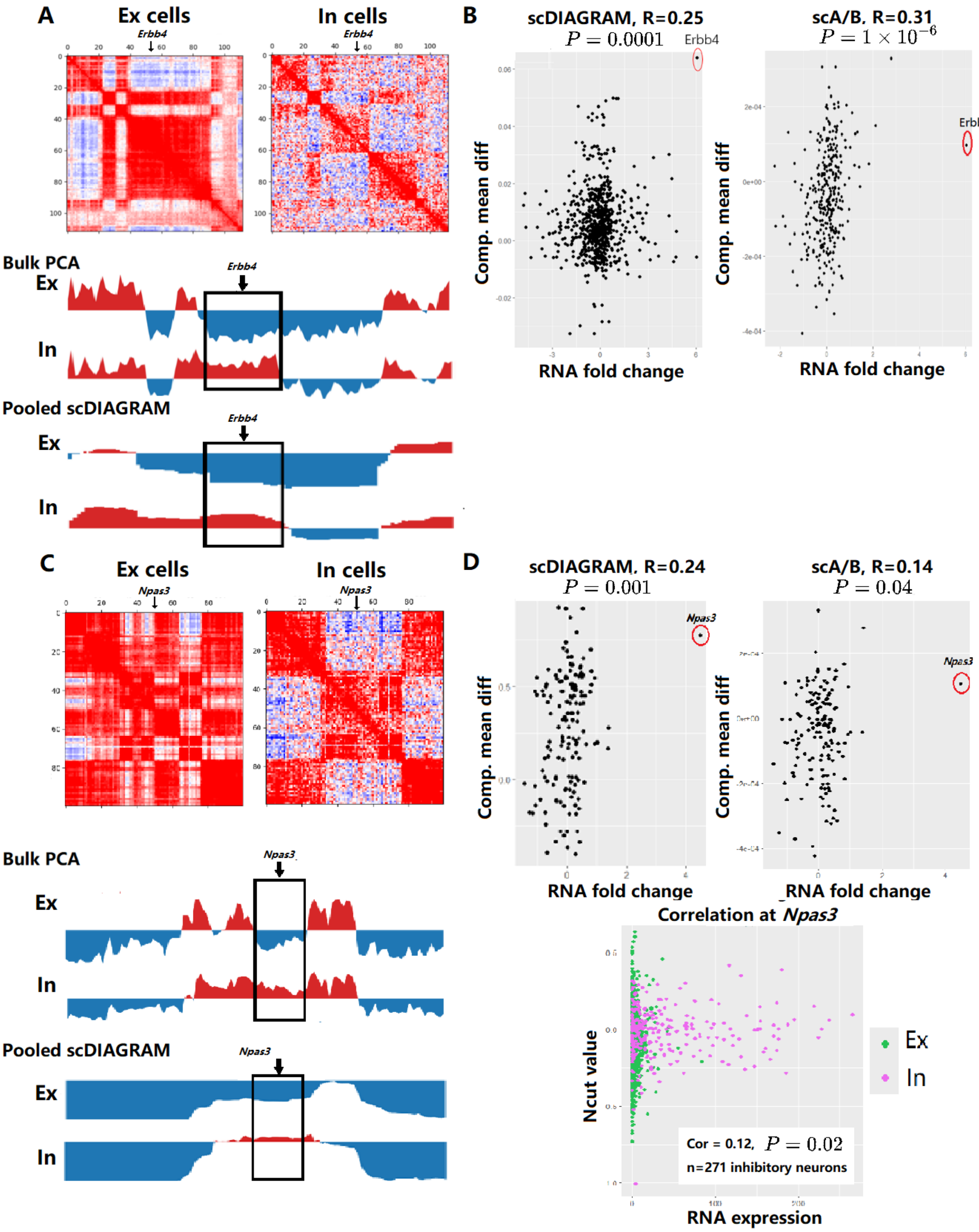

Supplementary Fig. S19. Compartment-RNA relationships at *Erbb4* and *Npas3* loci.

(A,C) Pseudo-bulk Hi-C matrices, bulk PCA, and pooled scDIAGRAM compartments at (A) *Erbb4* and (C) *Npas3* loci.

(B,D) Pearson's correlations between compartment differences (for scDIAGRAM and scA/B) and RNA log fold changes (In vs Ex) for (B) chr1 DEGs (n=230 genes, one-sided tests for nonzero correlations) and (D) chr12 DEGs (n=158 genes), with *Erbb4* and *Npas3*

showing the most significant increase in both RNA expressions and compartments (by scDIAGRAM). scA/B only exhibited moderate difference on these two loci, indicating scDIAGRAM was more consistent with RNA observations.

(D) The correlation between RNA expressions and real-valued scCompartments (from scDIAGRAM) at this locus across single cells.

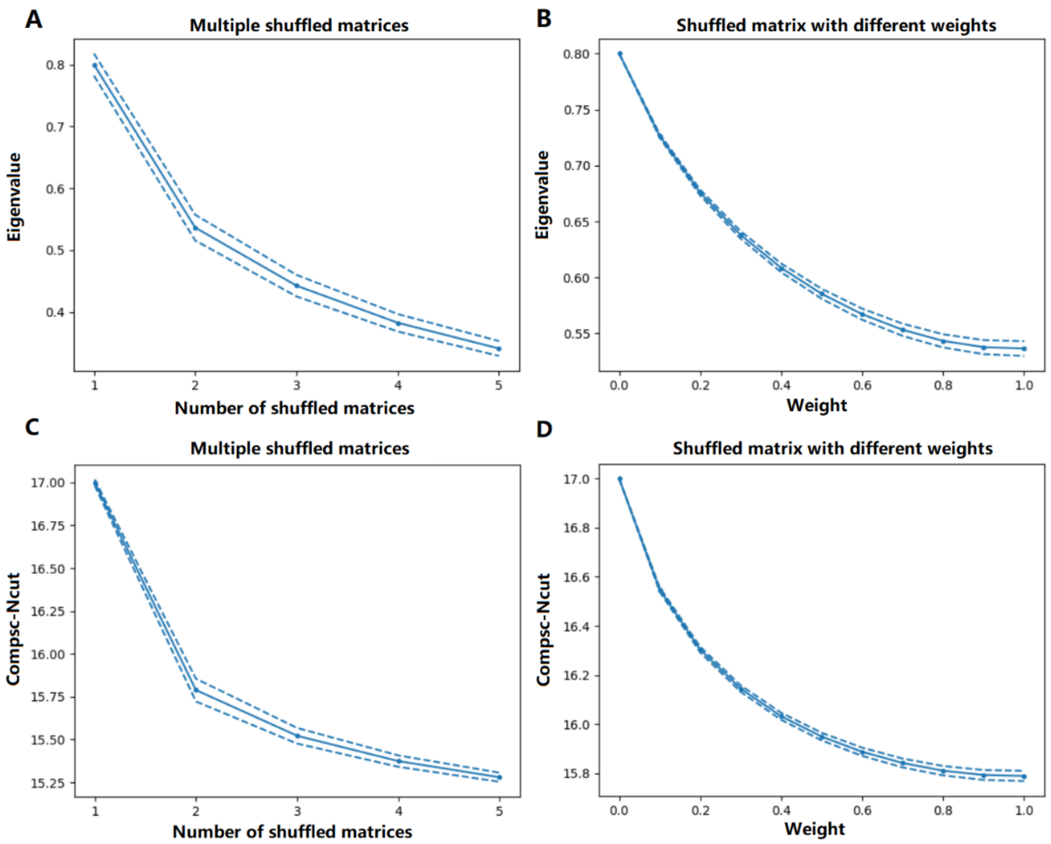

Supplementary Fig. S20. The eigenvalue and Compsc-Ncut in the permutation experiments.

(A-B) The second eigenvalue changes when:

(A) pooling original data with K=1-5 shuffled matrices, or

(B) pooling original data with a single weighted shuffled matrix (0.1-0.9 weights).

(C-D) Corresponding Compsc-Ncut changes under the same conditions.

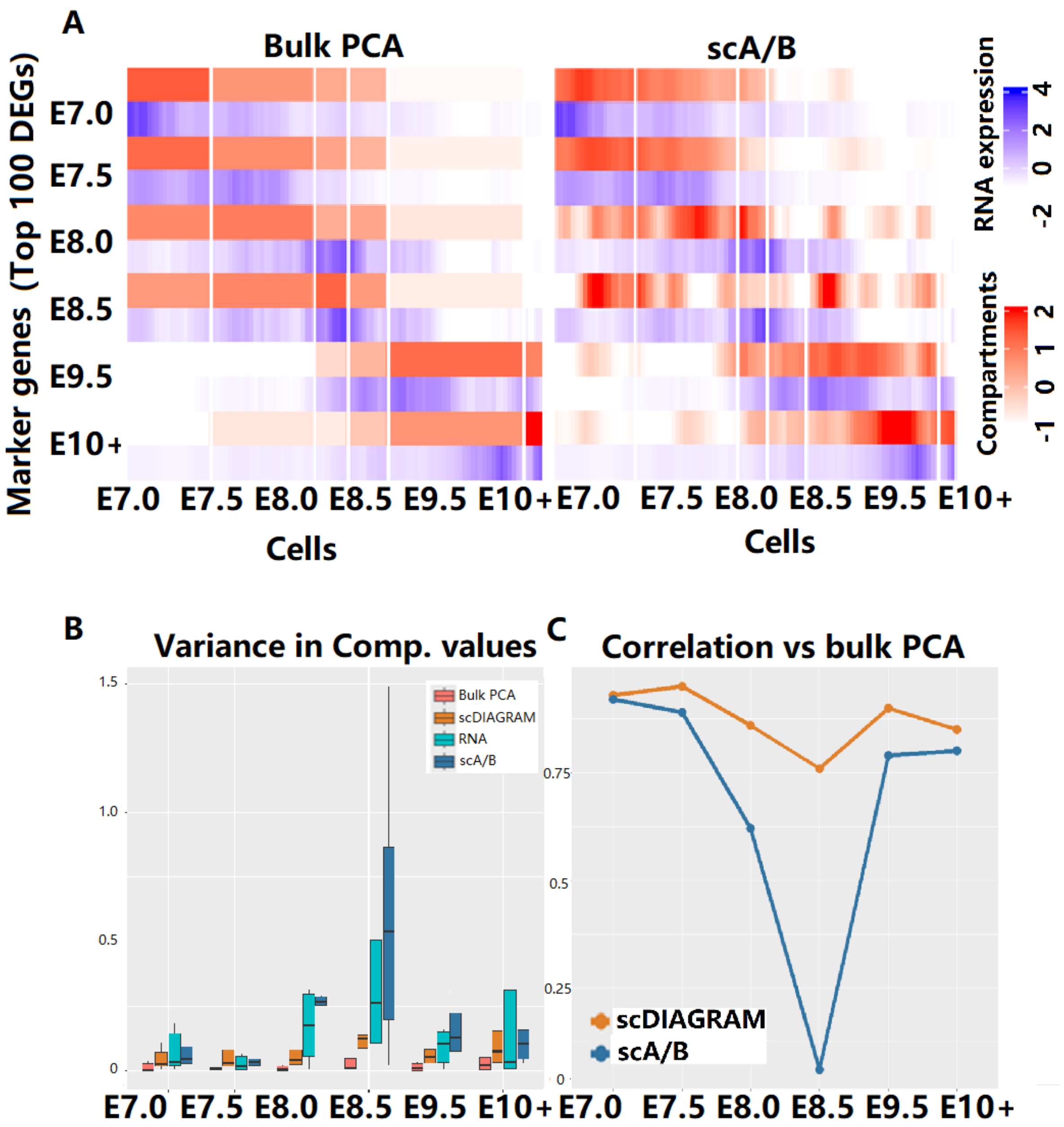

Supplementary Fig. S21. Compartment dynamics during embryo development (E7.0-E10+).

(A) Stage-specific marker gene enrichment (pseudo-time ordered) of compartments from bulk PCA and scA/B. Bulk PCA was calculated from pseudo-bulk matrices at each stage. The top 100 marker genes (ordered by fold change) at each stage were used.

(B) Compartmental variances at each stage for different methods.

(C) scDIAGRAM correlated more closely with bulk PCA than scA/B (row-wise correlations for heatmaps in (A) and Fig. 4C in the maintext).

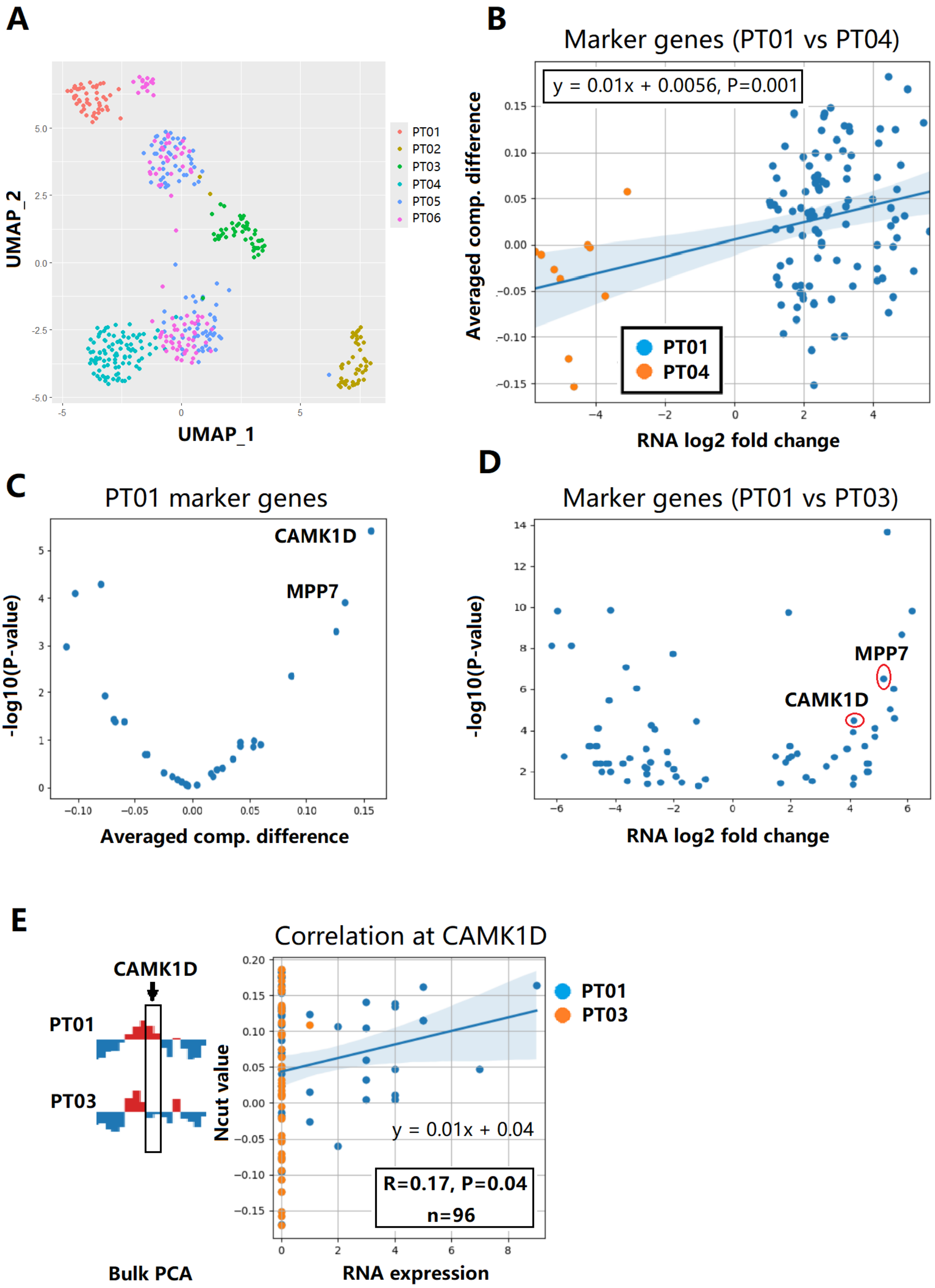

Supplementary Fig. S22. Compartmental differences in AML.

(A) UMAP embedding of scRNA-seq data showed patient-specific clustering, motivating our focus on compartmental differences between patients.

(B) Correlation between changes in compartmentalization and gene expression for marker genes comparing patients PT01 and PT04.
(C) Volcano plot showing differential scDIAGRAM compartment values between PT01 and PT03. P-values were calculated using a two-sample t-test. Only marker genes from PT01 are shown.
(D) Volcano plot of differential gene expression between PT01 and PT03, with P-values computed using the MAST test in Seurat.
(E) Visualization of bulk PCA signals at the CAMK1D locus in PT01 and PT03, along with RNA expression and scDIAGRAM Ncut values for each single cell at the same locus.

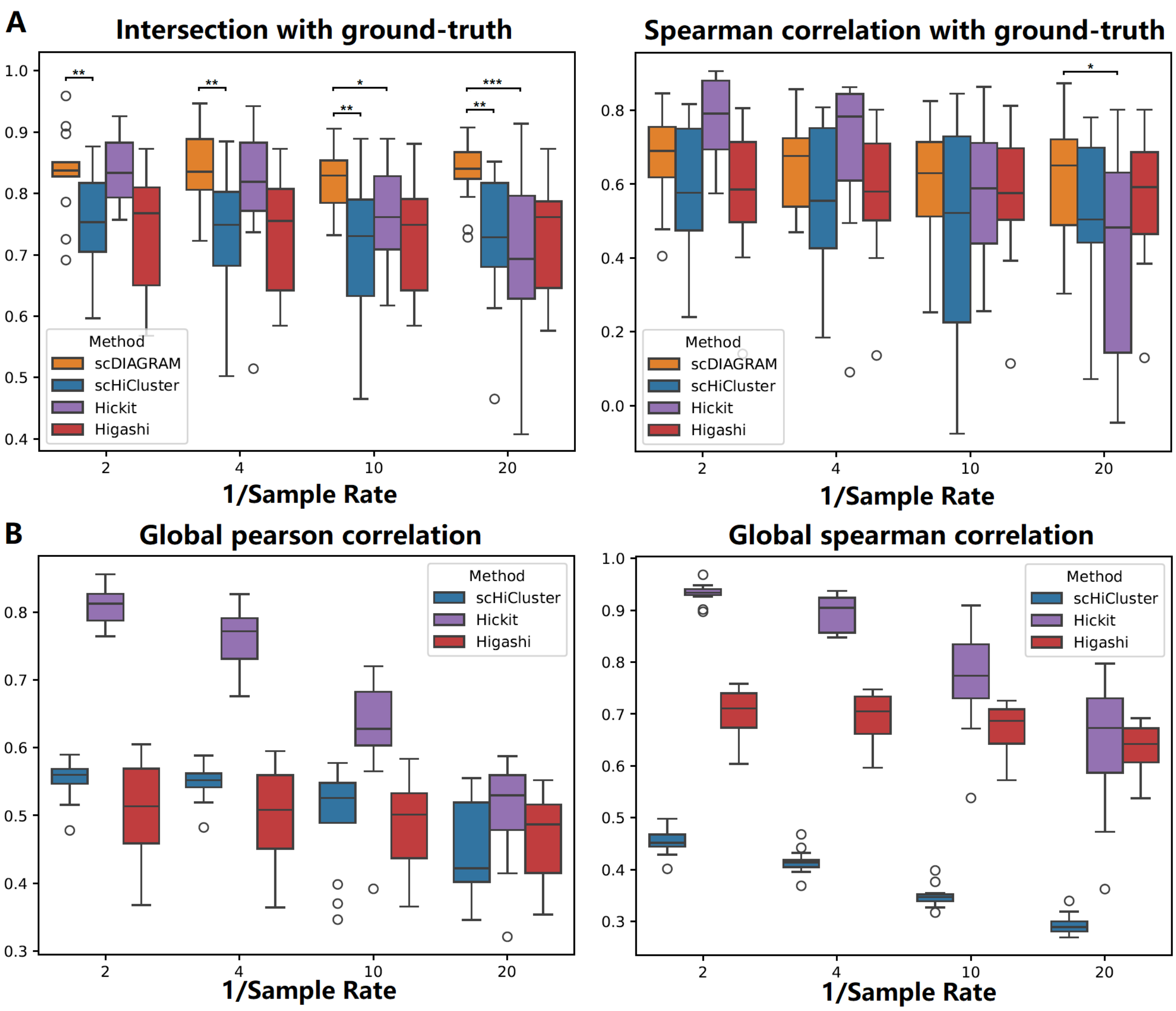

Supplementary Fig. S23. Performance on downsampled high-coverage scHi-C (the DipC data).

(A) Using Hickit (rate=1) as ground truth (n=14 GM12878 cells in total, chr1, at 500kb resolution), scDIAGRAM outperformed scHiCluster and Higashi at all sampling rates. It performed better than Hickit at lower rates where Hickit performance declined.

(B) Global Pearson/Spearman correlations (the correlation between two flattened Hi-C matrices) with the ground-truth showed imputation methods (scHiCluster, Higashi, Hickit) produced different but increasingly similar imputation matrices when the sample rate decreased. Higashi demonstrating the greatest robustness to downsampling.

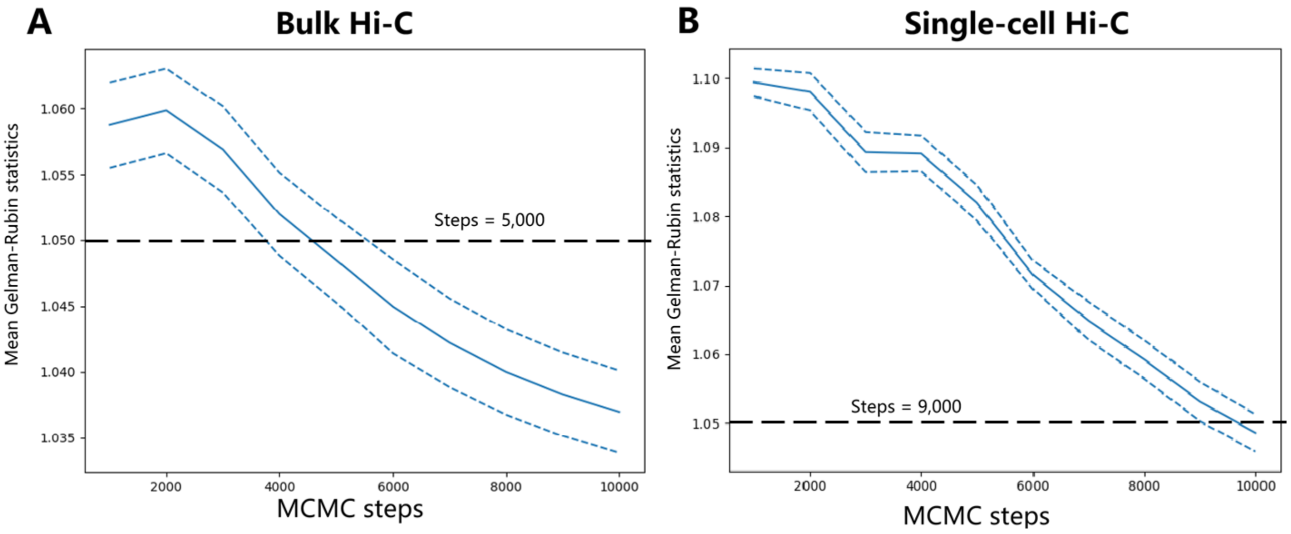

Supplementary Fig. S24. Diagnosis of MCMC convergence.
The averaged Gelman-Rubin statistics were computed for 20 CPs at 1 Mb resolution for both bulk (A) and single-cell (B) Hi-C matrices. For bulk data, the statistics dropped below 1.05 after 5,000 steps, whereas single-cell data required 9,000 steps, reflecting the higher noise level in scHi-C. In this study, we fixed MCMC steps at 10,000 or 20,000 depending on the dataset. The figure demonstrates that MCMC had already converged at these steps. All MCMC runs were repeated five times to ensure reproducibility. Error bar were computed from the standard deviations of the statistics for 20 CPs.

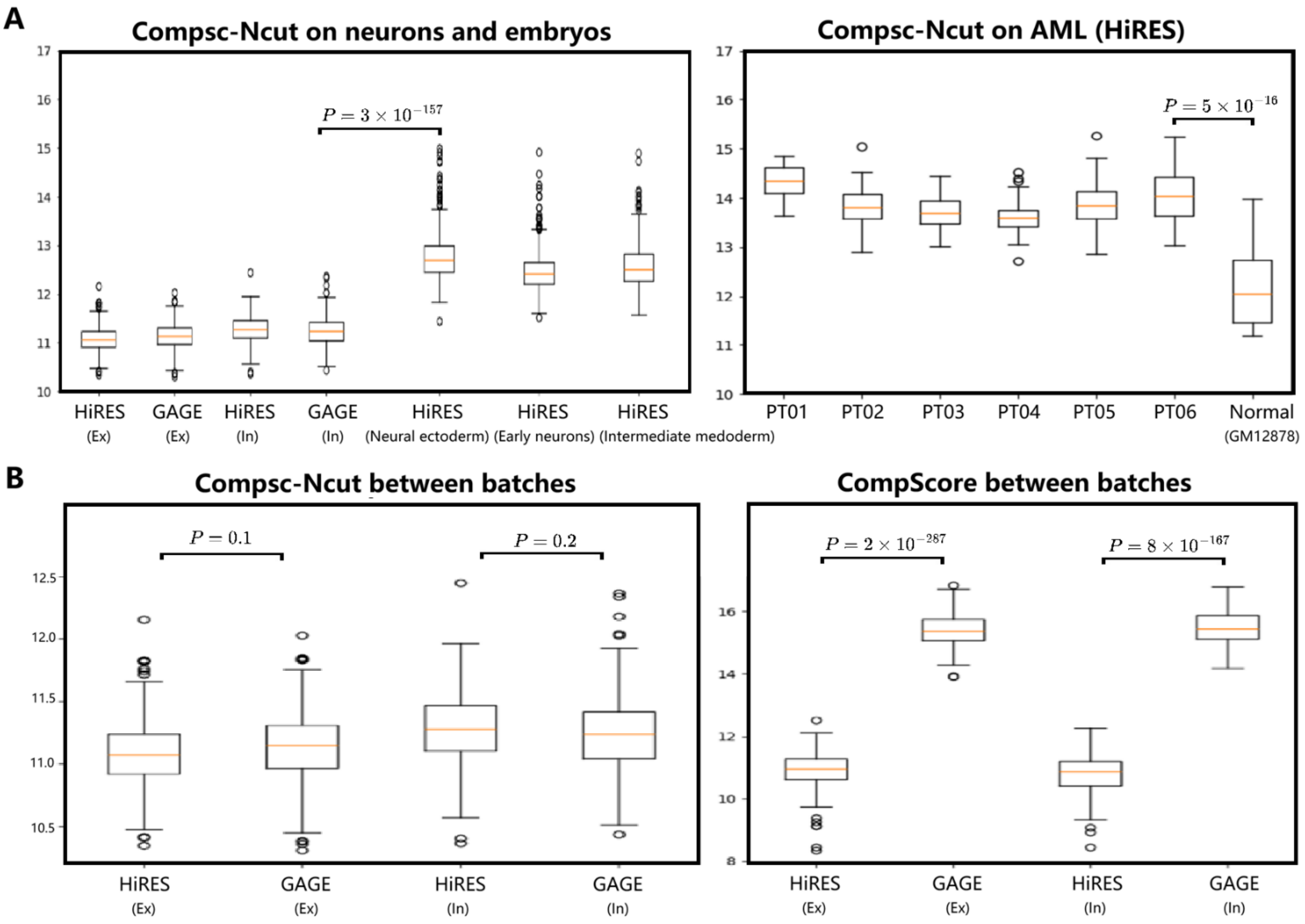

Supplementary Fig. S25. Comparison of Compsc-Ncut across different datasets.

(A) Compsc-Ncut was applied to datasets including neuron cells, embryonic development, and the AML dataset presented in this study. Both the developmental and AML datasets exhibited higher compartmental strength.

(B) Compsc-Ncut and standard CompScore were compared on adult mouse neuron cells from different labs (HiRES and GAGE-seq). Compsc-Ncut showed no detectable batch effect for the same cell type, whereas CompScore still exhibited substantial batch effects.

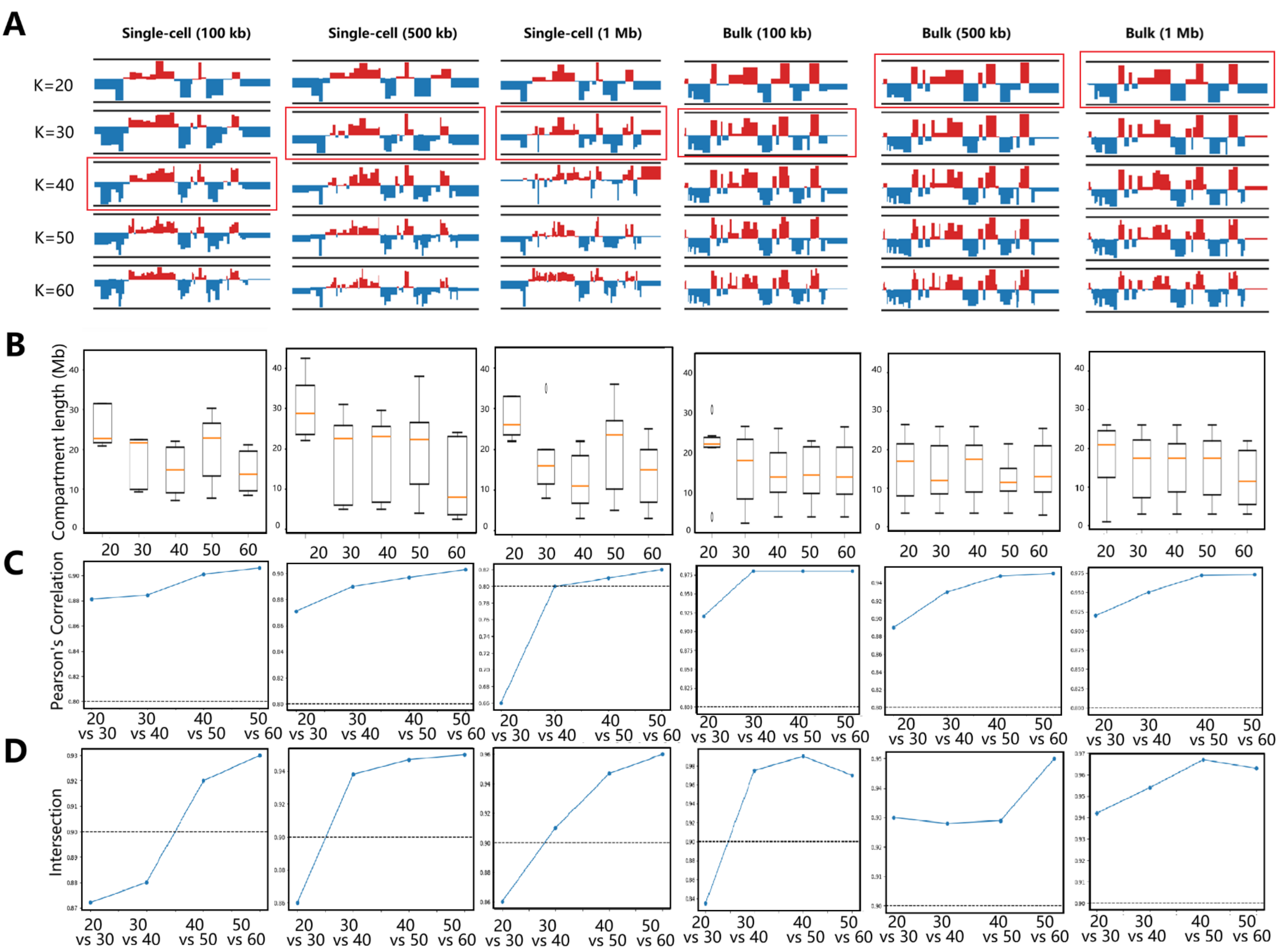

Supplementary Fig. S26. The relation between the CP number K, the compartmental lengths. data resolution and noise level.

Here we tested scDIAGRAM with various CP number K, at different resolutions (100kb, 500kb, 1Mb) and different noise level (comparing a single-cell and a bulk data from the same AML subtype, on chr1 after the centromere). Overall, these compartments were consistent. The selected K for each data was highlighted with a red box (A). We also computed the compartmental length (B), the Pearson's correlation/intersection (C, D) we used to choose K in the 2nd-4th panel. We argued data with higher resolution (100 kb vs 500 kb and 1 Mb) and larger noise level (single-cell vs bulk) needed more CP numbers. Larger CP numbers might output compartments with smaller lengths. The compartmental lengths were little affected by the resolution and noise level used, at least in this dataset. This further strengthened the biological usage of A/B comparmtents.

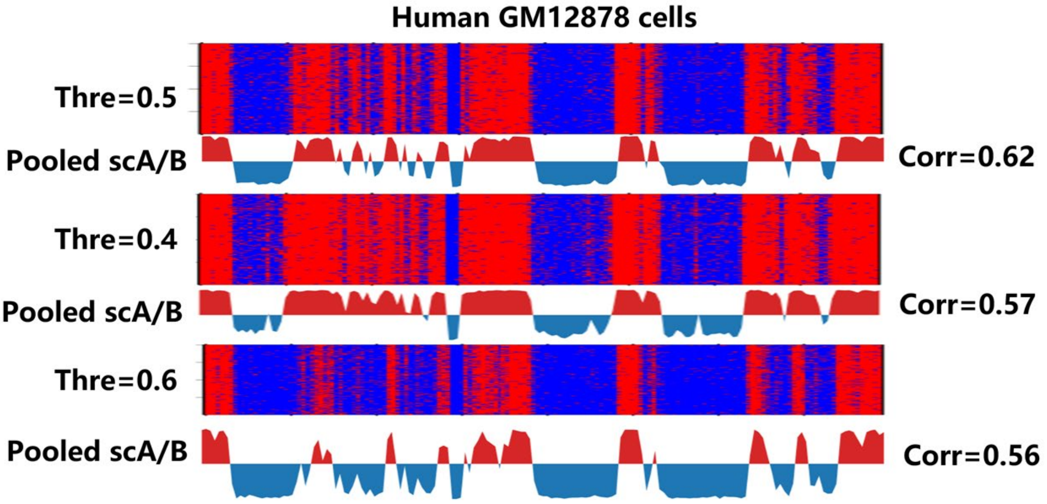

Supplementary Fig. S27. Robustness of scA/B to the threshold used for partitioning. The threshold for dividing loci into two groups using scA/B was varied around 0.5 (the value used in our study). Both the single-cell heatmaps and pooled scA/B compartments remained consistent across thresholds. Pearson correlations between the pooled scA/B and bulk PCA were also computed. Since scA/B values were normalized to [0,1], a threshold of 0.5 was a natural choice, which also yielded the highest correlation.

Supplementary Table S1. Clinical and molecular characteristics of the AML samples. This table provided all information to define the AML subtype.

Abbreviations: F, female; M, male; FAB, French-American-British classification; WT, wild-type; Mut, mutant; Pos, positive (for FLT3-ITD, present); Neg, negative (for FLT3-ITD, absent).

The following are the details for each clinical or molecular characteristics.

Blast count: The blast percentage was determined by microscopic examination of Wright-Giemsa-stained bone marrow aspirate slides at diagnosis. This value represents the percentage of myeloblasts among at least 200 nucleated cells in the bone marrow.

Cytogenetics: This value was performed on bone marrow samples at diagnosis using G-banding technique (Giemsa staining). Karyotypes were described according to the International System for Human Cytogenetic Nomenclature (ISCN).

NPM1 mutaion: This value was assessed by real-time quantitative PCR for mutant types A, B, and D. Mutations were reported as mutant or wild-type. For mutant cases, the variant allele frequency was calculated as the ratio of mutant to control gene (ABL) copy number.

FLT3-ITD status: This value was assessed by PCR-capillary electrophoresis. Mutations were reported as positive (with indicated variant allele frequency) or negative. The allelic ratio (AR) was calculated as the peak height (or area) of the mutant ITD allele divided by the peak height (or area) of the wild-type FLT3 allele. Additionally, the mutant burden was estimated as the percentage of the ITD mutant peak area relative to the total peak area (wild-type + ITD), representing the approximate proportion of cells harboring the ITD mutation in the sample.

TP53 mutation: This value was assessed by Sanger sequencing of cDNA covering exons 4 through 10. Mutations were reported as mutant or wild-type.

SRSF2 mutation: This value was assessed by Sanger sequencing of the SRSF2 genomic regions. Mutations were reported as mutant or wild-type.

Expression status: High expression was defined as a transcript level exceeding the established normal cutoff (0.6% for WT1, 8.0% for EVI1and 0.1% for MLL-PTD, normalized to ABL copies) as per the clinical laboratory's quantitative RT-PCR assay.

Fusion gene detection: The presence of fusion genes was assessed by a multiplex quantitative RT-PCR assay covering all major subtypes. A patient was classified as “fusion-negative” only if all tested fusion transcripts were reported as “not detected” (typically denoted as “0”). The major subtypes include BCR::ABL (P210, P190, P230, Variants), RUNX1::RUNX1T1 (AML1::ETO), CBFB::MYH11, PML::RARA (Long form-L, Variable form-V, Short form-S), KMT2A (MLL) rearrangements (e.g., with AF4, AF6, AF9, AF10, ELL, ENL, AF1p, AF1q, SEPT5, SEPT6, SEPT9, SEPT11, AF17, TET1), and other rare fusions (e.g., PICALM::AF10, AML1::MDS1, TLS::ERG, DEK::NUP214, SET::NUP214, TEL::PDGFRB, RBM15::MKL1, NUP98::HOXA9, NUP98::NSD1).

Sample	Tissue	Gender	Age	FAB	Blast count	Cytogenetics	NPM1 mutaion	FLT3-ITD status	WT1 expression status	EVI1 expression status	MLL-PTD expression status	Fusion gene detection	Patient status	Precited WHO-HAEM5 (2022) Classification	Predicted ICC (2022) Classification	Predicted ELN (2022) Classification	Remarks
**PT01**	Bone marrow	F	63	M4	30%	46,XX,t(4:9)(q25:q22)[1]/46,XX[34]	Mut (VAF 22.93%)	Neg	High (25.1%)	Normal (0.090%)	Normal (0.048%)	Neg	Newly diagnosed AML, pre-treatment.	AML with mutated NPM1	AML with mutated NPM1	Favorable	TP53 WT.
**PT02**	Bone marrow	M	56	M2	25%	46,XY[30]	WT	Neg	High (5.9%)	High (49.8%)	Normal (0.091%)	Neg	Newly diagnosed AML, pre-treatment.	AML, defined by differentiation (M2 type)	AML, not otherwise specified (M2 type)	Cannot be classified	
**PT03**	Bone marrow	M	37	M2	42%	46,XY[20]	WT	Neg	High (4.9%)	Normal (0.075%)	Normal (0.025%)	Neg	Newly diagnosed AML, pre-treatment.	AML, defined by differentiation (M2 type)	AML, not otherwise specified (M2 type)	Intermediate	TP53 WT.
**PT04**	Bone marrow	F	46	M2	95%	46,XX[19]	Mut (VAF 56.31%)	Pos (AR 1.73)	High (38%)	Normal (0.12%)	Normal (0.0089%)	Neg	Newly diagnosed AML, pre-treatment.	AML with mutated NPM1	AML with mutated NPM1	Adverse	TP53 WT.
**PT05**	Bone marrow	F	21	M2	20%	46,XX[20]	WT	Neg	High (39.3%)	Normal (0.78%)	Normal (0.028%)	Neg	Newly diagnosed AML, pre-treatment.	AML, defined by differentiation (M2 type)	AML, not otherwise specified (M2 type)	Intermediate	TP53 WT; SRSF2 WT; KRAS,NRAS mutations detected by NGS.
**PT06**	Bone marrow	M	56	M4	65%	46,XY[20]	WT	Neg	High (47.2%)	Normal (1.6%)	Normal (12.4%)	Neg	Newly diagnosed AML, pre-treatment.	AML, defined by differentiation (M4 type)	AML, not otherwise specified (M4 type)	Cannot be classified	TP53 WT; SRSF2 WT; DNMT3A, FLT3-ITD, and IDH2 mutations detected by NGS.
